# Levels and instability of transthyretin and correlations with core biomarkers in Alzheimer’s disease

**DOI:** 10.1038/s41598-026-41717-7

**Published:** 2026-03-12

**Authors:** Tiago Gião, Miguel Tábuas-Pereira, Inês Baldeiras, Joana Saavedra, Alexandre Dias, Maria João Saraiva, Maria Rosário Almeida, Isabel Santana, Isabel Cardoso

**Affiliations:** 1 i3S - Instituto de Investigação e Inovação em Saúde, Universidade do Porto, 4200- 135 Porto, Portugal; 2 IBMC - Instituto de Biologia Molecular e Celular, Universidade do Porto, 4200-135 Porto, Portugal; 3 ICBAS - Instituto de Ciências Biomédicas Abel Salazar, Universidade do Porto, 4050- 313 Porto, Portugal; 4 Faculdade de Medicina, Universidade de Coimbra, 3000-075 Coimbra, Portugal; 5 CIBB - Centro de Inovação em Biomedicina e Biotecnologia, Universidade de Coimbra, 3004-504 Coimbra, Portugal; 6 CNC - Centro de Neurociências e Biologia Celular, Universidade de Coimbra, 3004- 504 Coimbra, Portugal; 7 Departmento de Neurologia, Hospital da Universidade de Coimbra, ULS de Coimbra, 3004-561 Coimbra, Portugal; 8 Ipatimup - Instituto de Patologia e Imunologia Molecular, Universidade do Porto, 4200-135 Porto, Portugal

**Keywords:** Transthyretin, Alzheimer’s disease, Amyloid-β, MCI, Instability, Blood-based biomarkers, Biochemistry, Biological techniques, Molecular biology, Neuroscience, Biomarkers, Diseases

## Abstract

Transthyretin (TTR) has gained attention due to its implication in Alzheimer’s Disease (AD) by modulating amyloid-beta (Aβ) pathology. This study aimed to establish associations between TTR and key AD biomarkers, to enhance our understanding of the role of TTR in AD pathogenesis. We evaluated TTR levels and tetrameric instability in plasma and CSF in mild cognitive impairment (MCI-AD) and Dementia-AD patients examining associations with clinical, biochemical and genetic data. Plasma TTR levels were lower in Dementia-AD, especially in women. In the MCI-AD group, CSF TTR levels inversely correlated with markers of amyloid pathology and neurodegeneration such as Aβ40, p-Tau181, t-Tau and NfL. CSF TTR instability was negatively associated with CSF Aβ42, indicating an association with increased amyloid burden. In the whole cohort, plasma TTR instability correlated with CSF TTR instability. In line with the strong association between CSF TTR instability and Aβ42, we showed increased TTR instability in samples incubated with Aβ42. No significant associations were observed in the Dementia-AD group. This study emphasizes that TTR is associated with Aβ clearance and neurodegeneration. We propose Aβ42 and its increase in the brain as a factor destabilizing the TTR tetrameric fold, impairing TTR function.

**Supplementary Information:**

The online version contains supplementary material available at 10.1038/s41598-026-41717-7.

## Introduction

Mild cognitive impairment (MCI) represents a transitional stage within the clinical progression of Alzheimer’s Disease (AD) and is frequently accompanied by early neuropathological changes^1,2^. Because individuals with MCI already show a significant risk of progression to dementia, this stage has become a major focus for dementia prevention and early diagnosis research. Recent meta-analytic evidence indicates that approximately 15.6% of community-dwelling adults aged 50 years and older meet the criteria for MCI^3^, and that conversion rates from MCI to dementia range between 10% and 13% annually^4–7^. Most of these patients already exhibit neuropathological hallmarks of AD, supporting the concept of MCI-AD as a prodromic or pre-dementia state of dementia due to AD (Dementia-AD) in the clinical continuum of AD.

One of the main challenges in AD research is the identification of early and accessible biomarkers that accurately reflect underlying pathology. Although current treatments do not reverse pathological changes, early diagnosis and intervention may delay cognitive decline^8,9^.

Core AD biomarkers, such as total tau (t-tau), Phosphorylated-tau at threonine 181 (p-tau181), amyloid-β 42 (Aβ42), amyloid-β 40 (Aβ40), and the Aβ42/Aβ40 ratio, measured in cerebrospinal fluid (CSF), have greatly improved diagnostic accuracy^10,11^. Recently, plasma-based biomarkers such as Aβ42, Aβ40, p-Tau181, p-Tau217, t-Tau, neurofilament light chain (NfL), and glial fibrillary acidic protein (GFAP) have gained attention as less invasive and more accessible tools for disease monitoring^12^. Despite these advances, complementary biomarkers that capture additional molecular processes underlying AD pathogenesis are still needed. In this context, transthyretin (TTR), a protein known to interact with Aβ and modulate its metabolism, has emerged as a promising candidate biomarker in AD.

TTR is a homotetrameric protein that transports thyroxine (T4) and retinol-binding protein in both blood and CSF. It is primarily synthesized in the liver and choroid plexus^13^, constituting approximately 5% of total CSF proteins and less than 1% of plasma proteins^14,15^. Beyond its transport function, TTR acts as a neuromodulator^16,17^ and contributes to memory preservation during aging^18,19^.

In the 1990s, Schwarzman and colleagues identified TTR as the primary Aβ-sequestering protein in CSF^20^. It is hypothesized that TTR binds to Aβ, preventing its aggregation and toxicity, and thereby exerts a protective role against AD pathology^21–26^. In vivo studies further support this neuroprotective effect in AD. In AD mouse models, reduced TTR expression increases Aβ deposition^27,28^, whereas TTR overexpression decreases amyloid burden and improves memory function^29^. At the vascular level, TTR promotes Aβ clearance from the brain across the blood-brain barrier (BBB)^30^, and its absence is associated with vascular impairment^31^.

Despite these protective functions, clinical data in humans remain inconsistent. Several studies report lower plasma/serum TTR levels in AD compared to controls^32–34^, while reports on CSF TTR levels are more conflicting. The mechanisms underlying this reduction are unclear but may involve tetrameric instability, which accelerates TTR clearance^35^ and reduces its Aβ-binding capacity^21,36,37^.

In this study, we evaluated TTR levels and tetrameric instability in both plasma and CSF of MCI-AD and Dementia-AD patients to monitor changes along disease progression. We further assessed their association with established AD biomarkers (Aβ, Tau, NfL, and GFAP) and clinical variables. We hypothesized that lower TTR levels and higher instability would be associated with greater amyloid pathology and neurodegeneration, supporting a role for TTR dysfunction in AD progression.

## Methods

### Subjects

This study included a cohort of 66 subjects (29 MCI-AD and 37 Dementia-AD patients) that were recruited at the Neurology Department of Coimbra University Hospital (HUC), Coimbra, Portugal. Patients were diagnosed in accordance with standard criteria and based on CSF-AD biomarkers: the framework for MCI due to AD and Dementia due to AD, proposed by NIA-AA criteria^38,39^. The baseline study and follow-up protocol have already been published elsewhere^40,41^.

Patients enrolled had biannual clinical observations and annual neuropsychological and functional evaluations. All patients underwent a thorough biochemical, neurological and imaging (CT, MRI and SPECT) evaluation and performed a lumbar puncture for CSF-AD biomarkers determination and genetic studies (APOE). Amyloid-PET and mutation analysis were more restricted, although considered respectively in patients with inconclusive CSF-biomarkers and younger patients. At baseline, a neurologist completed a medical history with the patient and the caregiver, and conducted a general physical, neurological and psychiatric examination as well as a comprehensive diagnostic battery protocol, including: cognitive instruments such as the Mini Mental State Examination (MMSE)^42^ Portuguese version^43^, The Montreal Cognitive Assessment (MoCA)^44^ Portuguese version^45^, the Alzheimer Disease Assessment Scale—Cognitive (ADAS-Cog)^46,47^ Portuguese version^48^ and a comprehensive neuropsychological battery with normative data for the Portuguese population (Lisbon Battery for Dementia Assessment (BLAD))^49^ exploring memory (Wechsler Memory Scale subtests) and other cognitive domains (including language, praxis, executive functions and visuoconstructive tests); and standard staging scales which provide objective information about subject performance in various domains, including the Clinical Dementia Rating scale (CDR)^50^ for global staging, the Disability Assessment for Dementia (DAD)^51,52^ for evaluation of functional status and the Neuropsychiatric Inventory (NPI)^53,54^ to characterize the psychopathological profile, including the presence of depression. All of the available information (baseline cognitive test, staging scales, clinical laboratory and imaging studies) was used to reach a consensus research diagnosis, supported by CSF-AD biomarkers.

Dementia-AD participants were diagnosed according to the Diagnostic and Statistics Manual for Mental Disorders—fourth edition text review (DSM-IV-TR) criteria, and AD according to the 2011 NIA-AA criteria^39^. These cases were classified as probable AD dementia according to clinical, CSF-AD biomarkers and neuroimaging features already referred to. Severity of dementia (mild, moderate or severe) was based on the Clinical Dementia Rating scale (CDR)^50^ for global staging.

MCI patients included in this study were of the amnestic type, diagnosed in accordance with criteria defined by *Petersen et al.*^55^ and the framework for MCI due to AD proposed by NIA-AA criteria^38^. Petersen et al.’s criteria were operationalized as follows: a subjective complaint of memory decline (reported by the subject or an informant); an objective memory impairment (considered when scores on standard Wechsler memory tests were > 1.5 SDs below age/education-adjusted norms) with or without deficits in other cognitive domains; normal general cognition suggested by normal scores for the MMSE and MoCA using the Portuguese cutoff scores^45,56^; largely normal daily life activities, evaluated with a functional scale (DAD); and absence of dementia, indicated by a CDR rating of 0.5. All patients were in a stable condition, without acute comorbidities. In addition to classification based on clinical stage, participants were stratified by APOE genotype. This subgroup analysis aimed to determine if APOE ε4 influenced TTR levels and tetrameric stability. As exclusion criteria for enrollment of participants, besides being in an unstable condition namely by acute comorbidities, we considered a significant underlying medical or neurological illness revealed by laboratory tests or imaging besides AD; a relevant psychiatric disease, including major depression, suggested in the medical interview and confirmed by the GDS, CT or MRI demonstration of significant vascular burden^57^.

### Samples collection

CSF and blood samples were collected from patients as part of their regular clinical diagnostic evaluation. Pre-analytical and analytical procedures were done according to BIOMARKAPD guidelines for CSF-AD biomarkers^58^.

CSF Samples were collected in sterile polypropylene tubes, immediately centrifuged at 1800 g (10 min at 4 °C), aliquoted into polypropylene tubes and stored at − 80 °C until analysis.

Blood samples were collected into plasma separation and EDTA tubes on the same day as the lumbar puncture, centrifuged at 1800 g for 10 min at 4 °C, aliquoted into polypropylene tubes, and stored at −80 °C.

### Apolipoprotein E (APOE) genotyping

For Apolipoprotein E (APOE) genotyping, DNA was isolated from whole EDTA blood using a commercial kit (Roche Diagnostics GmbH, Mannheim, Germany), as described by the manufacturer. The analysis of the two polymorphisms at codons 112 and 158 of the APOE gene (rs429358 and rs7412) was performed by PCR-RFLP assay, as described previously^59^.

### Analyses of AD core biomarkers

CSF Aβ42, Aβ40, t-Tau and p-Tau181 were measured separately by commercially available fully automated chemiluminescence enzyme immunoassays (LUMIPULSE, Fujirebio, Japan), as previously described^60^. External quality control of the assays was performed under the scope of Alzheimer’s Association Quality Control Program for CSF Biomarkers^61^.

Previously determined laboratory specific cut-offs for LUMIPULSE^60^ were used to dichotomize markers as normal (-) or abnormal (+) and to classify samples according to the ATN scheme^62^: the Aβ42/Aβ40 ratio for evidence of amyloid deposition (A), p-Tau181 for evidence of Tau aggregation (T) and t-Tau for evidence of neurodegeneration (N). According to this scheme, all samples used on this study were classified as being A + T+N+, therefore as having biological AD.

### Analyses of NfL and GFAP

NfL and GFAP CSF levels determinations were done in duplicate, using different aliquots than the one used for CSF-AD biomarkers. For NfL, CSF was diluted 1:1 and NfL was quantified using an ELISA assay (NF-light; Uman Diagnostics, Sweden), as previously described^63^. For GFAP, samples were diluted 40 times and measurements were made by single molecule array in a SR-X platform (Quanterix) using the GFAP discovery kit (Quanterix) in accordance with the manufacturer’s instructions and sample type specifications.

### Analysis of the CSF/serum albumin quotient (QAlb)

Albumin plasma levels were determined in duplicate using bromocresol purple (BCP) Albumin Assay Kit (Sigma-Aldrich), while albumin CSF levels were measured by sandwich ELISA kits (Proteintech), both following the manufacturer’s instructions. The CSF and serum albumin from 66 paired samples were used to calculate the QAlb using the formula QAlb *=* [albumin (mg/mL)] CSF/[albumin (mg/mL)] plasma × 1000 and used as blood-brain barrier (BBB) measure.

### Transthyretin measurement

Plasma and CSF TTR levels were measured in duplicate by commercially available sandwich ELISA (Abcam), following the manufacturer’s instructions.

### Evaluating plasma and CSF TTR instability

To assess TTR instability, 4 µL of plasma or CSF was added to SDS reducing gel loading buffer (SDS final concentration = 0.5%) and samples were heated to 55 °C for 5 min. Proteins were then separated using 15% SDS-polyacrylamide gel electrophoresis (PAGE) and transferred to a PVDF membrane (Thermo Fisher) using a dry system (iBlot, Thermo Fisher). The membranes were then blocked for 1 h at room temperature with 5% powdered skimmed milk in PBS containing 0.05% Tween-20 (PBS-T). Immunoblotting was then performed using a mouse anti-TTR antibody^64^ to detect monomer and dimer TTR. The blots were developed using Clarity™ Western ECL substrate (Bio-Rad), and proteins were detected and visualized using a chemiluminescence detection system (ChemiDoc, Bio-Rad). Images were analyzed and band intensities were quantified using Image Lab (Bio-Rad, version 4.1). The functional conformation of TTR is the tetramer and here TTR instability was expressed as the monomer/dimer intensity ratio, used as a surrogate of tetramer dissociation, as described previously^37,65^.

### Preparation of soluble Aβ42

Synthetic Aβ42 (Genscript) was dissolved in hexafluoro-2-propanol (HFIP) (Sigma-Aldrich) and kept at RT for approximately 72 h. After, the HFIP was removed under a stream of nitrogen, and the powder was dissolved in DMSO at 2 mM. Soluble Aβ42 was obtained by diluting the peptide in Ham’s F12 medium. To confirm the presence of soluble Aβ42 species, samples were analyzed by transmission electron microscopy (TEM) and visualized by negative staining using uranyl acetate. Briefly, 5 µl aliquots were adsorbed into carbon coated collodion film supported on 300-mesh copper grids (Electron Microscopy Sciences, PA, USA) and negatively stained twice with 1% (m/v) uranyl acetate (Electron Microscopy Sciences, PA, USA). Grids were visualized with a JEOL (Tokyo, Japan) JEM1400 transmission electron microscope equipped with an Orious (CA, USA) Sc1000 digital camera and exhaustively observed.

### Assessment of tetrameric TTR instability when incubated with Aβ42

To assess the instability of tetrameric TTR in the presence of Aβ42, 10 µM of recombinant wild-type TTR (WT TTR, AlexoTech) was incubated at 37 °C overnight, either alone or with 40 µM of Aβ42 (Genscript), in duplicate. The samples were then subjected to semi-denaturing electrophoresis and Western Blot, as previously described for evaluating plasma and CSF TTR instability.

### Statistical analysis

Categorical variables were expressed as number (%) and compared with Chi-square test or Fisher’s Exact test, as appropriate. Continuous variables were expressed as mean (standard deviation) and compared using Student’s t-test or Mann-Whitney test, as appropriate.

For associations between TTR (levels and instability) and CSF AD biomarkers, linear regression models adjusted for age and sex were applied to estimate the regression coefficients (β). Interaction terms between TTR and clinical diagnosis (MCI-AD vs. Dementia-AD) were tested to assess stage-dependent effects. Correlations between TTR (levels and instability) and demographic variables or comorbidities (Supplementary Fig. 1) were also explored using Spearman’s correlation.

The statistical significance threshold was set at *P* < 0.05, two-sided. The analyses and figures were made using SPSS Statistics version 29 (IBM) and Graphpad Prism 8.

## Results

### Demographics and clinical description of the participants

A total of 66 participants with a biological diagnosis of AD supported by CSF biomarkers were included in this study, comprising 29 with mild cognitive impairment (MCI-AD) and 37 with dementia (Dementia-AD). Demographic and clinical information is summarized in Table 1, categorized by clinical staging MCI-AD versus Dementia-AD.

As expected, patients with Dementia-AD showed significantly lower cognitive performance compared to the MCI-AD group, indicated by lower MMSE scores (*p* < 0.001). In addition, Dementia-AD patients presented significantly reduced CSF Aβ42 and Aβ40 concentrations (both *p* < 0.001), consistent with a more advanced amyloid pathology. No significant differences were observed between the groups for CSF t-Tau, p-Tau181, NfL, or GFAP levels. Other demographic and clinical variables, including sex distribution, age, education, and APOE genotype were similar between groups.

Several comorbidities were documented among participants, with significant decreases observed in plasma and CSF glucose levels in Dementia-AD patients (Supplement, Table S1).

**Table 1:** Demographics and key characteristics of study participants.

Sex, M/F (% F)	MCI-AD (*n* = 29)	Dementia-AD (*n* = 37)	*P*
	10/19 (65.52)	15/22 (59.46)	0.615
Age (years)	68.69 (6.49)	68.92 (7.78)	0.771
Age onset (years)	66.38 (6.22)	64.35 (7.66)	0.335
Education (years)	7.34 (4.94)	6.62 (4.72)	0.262
MMSE score	23.80 (5.35)	13.68 (6.85)	< 0.001
APOE-ε2 carriers, n (%)	4 (13.79)	2 (5.41)	0.392
APOE-ε4 carriers, n (%)	14 (48.27)	20 (54.05)	0.641
ARWMC scale - WM	0.79 (0.85)	1.11 (1.02)	0.222
ARWMC scale - BG	0.41 (0.56)	0.59 (0.76)	0.429
CSF Aβ42 (pg/ml)	656.79 (183.67)	452.32 (159.22)	< 0.001
CSF Aβ40 (pg/ml)	13154.517 (3085.45)	9958.76 (3572.95)	< 0.001
CSF Aβ42/40 ratio	0.050 (0.008)	0.047 (0.010)	0.06
CSF t-Tau (pg/ml)	767.62 (335.71)	732.51 (303.28)	0.651
CSF p-Tau181 (pg/ml)	135.46 (62.54)	122.11 (49.31)	0.394
CSF NfL (pg/mL)	1485.99 (932.37)	1598.87 (603.42)	0.112
CSF GFAP (pg/mL)	29450.35 (10422.43)	29196.39 (10034.12)	0.964
Qalb	2.07 (0.59)	2.18 (0.86)	0.583

Differences between groups were assessed using Mann-Whitney U for continuous variables and chi-square test (for sex and ε4 carriers) or Fisher’s exact test (for APOE ε2) for categorical variables. Data are presented as mean (standard deviation) or number of participants (percentage), as appropriate. P values < 0.05 were considered statistically significant.

*Aβ* Amyloid-β, *AD* Alzheimer’s disease, *APOE* Apolipoprotein E, *ARWMC * Age-related white matter changes, BG Basal ganglia, *CSF* Cerebrospinal fluid, *GFAP* Glial fibrillary acidic protein, *MCI * Mild cognitive impairment, *MMSE* Mini-Mental state examination, *NfL* Neurofilament light chain, *p-Tau * Phosphorylated Tau, *SD* Standard deviation, *t-Tau* Total Tau, WM White matter.

### Evaluation of CSF and plasma TTR levels and instability

**Fig. 1:**
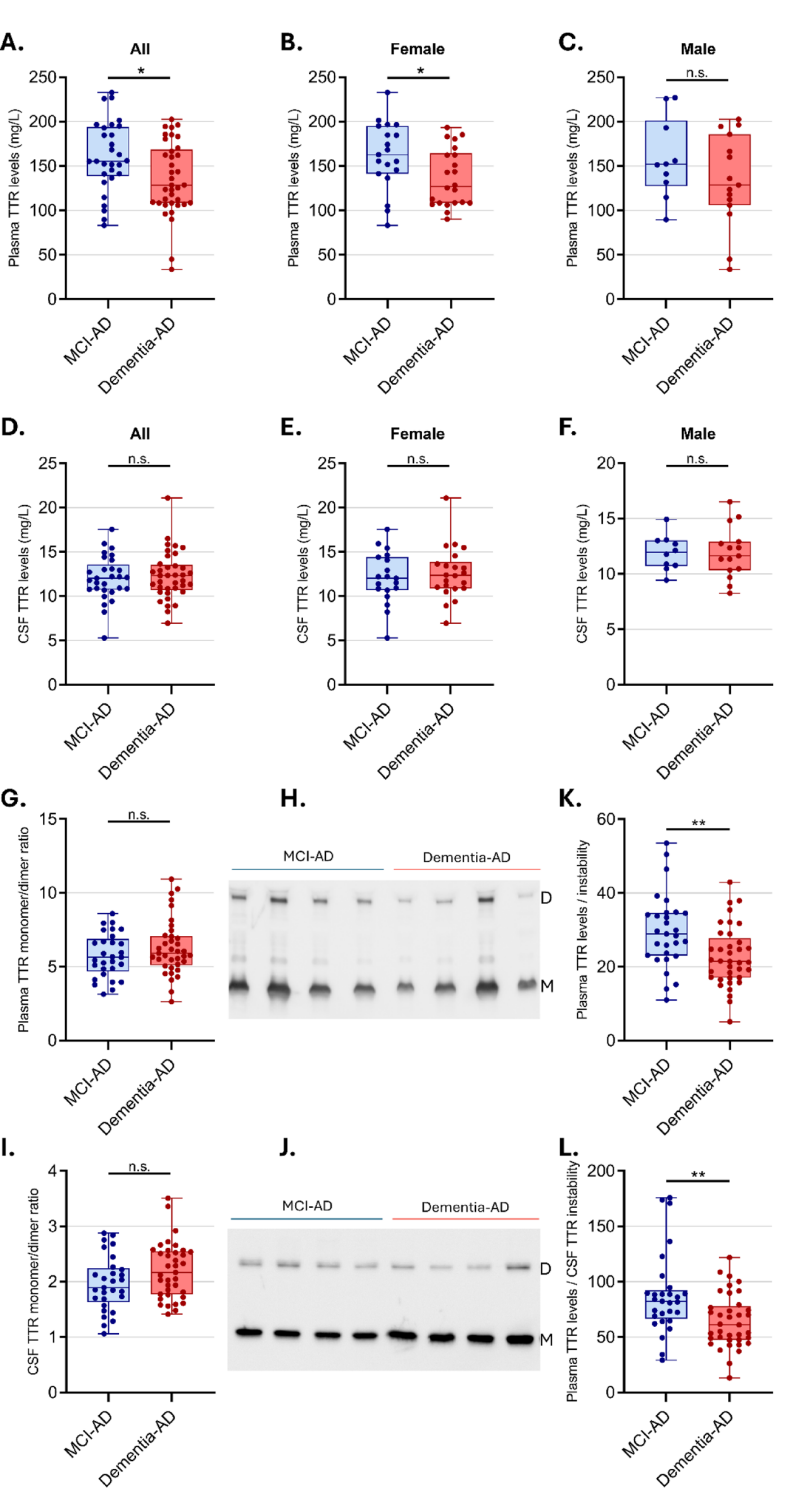
Evaluation of TTR levels and instability in plasma and CSF according to clinical diagnosis. Box-plots comparing plasma TTR levels in (**A**) MCI-AD (n=29) and Dementia-AD (n=37) groups; (**B**) MCI-AD (n=19) and Dementia-AD (n=22) in females; and (**C**) MCI-AD (n=10) and Dementia-AD (n=15) in males. Box-plot comparing CSF TTR levels in (**D**) MCI-AD (n=29) and Dementia-AD (n=37) groups; (**E**) MCI-AD (n=19) and Dementia-AD (n=22) in females; and (**F**) MCI-AD (n=10) and Dementia-AD (n=15) in males. Box-plots comparing TTR instability in (**G**) plasma and (**I**) CSF, in MCI-AD (n=29) and Dementia-AD (n=37) groups, by measuring the monomer/dimer ratio. Western blot of TTR in (**H**) plasma and (**J**) CSF samples from the groups analyzed, exhibiting monomer (M) and dimer bands (D) used for analysis of TTR instability. The ratio between plasma TTR levels and TTR instability in (**K**)plasma and (**L**)CSF. The boxplots depict the median (horizontal line within each box), interquartile range (IQR, box ends) and 1.5 × IQR (whiskers). Asterisks indicate significant differences, where * *p* < 0.05 by two-tailed Mann-Whitney test.

Plasma TTR levels were measured, revealing lower levels in Dementia-AD subjects compared to those with MCI-AD (*p* = 0.024) (Fig. 1 A). Stratification by gender showed that this difference was predominantly influenced by females, who exhibited a similar pattern of lower TTR levels in Dementia-AD versus MCI-AD patients (*p* = 0.036) (Fig. 1B), while no significant differences were observed among males (Fig. 1C). CSF analysis revealed no significant differences in TTR levels between MCI-AD and Dementia-AD groups (Fig. 1D), even when divided by gender (Fig. 1E, F).

We also quantified TTR instability, defined as the monomer/dimer ratio obtained from semi-denaturing immunoblots, reflecting the degree of tetramer dissociation. Although no significant differences were found between MCI-AD and Dementia-AD groups in either fluid, there was a trend indicating that CSF TTR may be less stable in Dementia-AD patients (Fig. 1G–J).

Interestingly, when we examined the ratios of plasma TTR levels to either plasma (Fig. 1K) or CSF TTR instability (Fig. 1L), we found significant differences. These findings suggest that TTR concentration and tetramer stability across compartments may be interrelated, potentially reflecting the dynamic role of TTR in the pathophysiology of AD.

### Association between TTR levels and instability with CSF biomarkers of AD

To investigate whether the relationship between TTR and AD-related biomarkers differs according to disease stage, we fitted linear models adjusted for age and sex and included an interaction term between TTR and clinical diagnosis (MCI-AD vs. Dementia-AD).

**Fig. 2:**
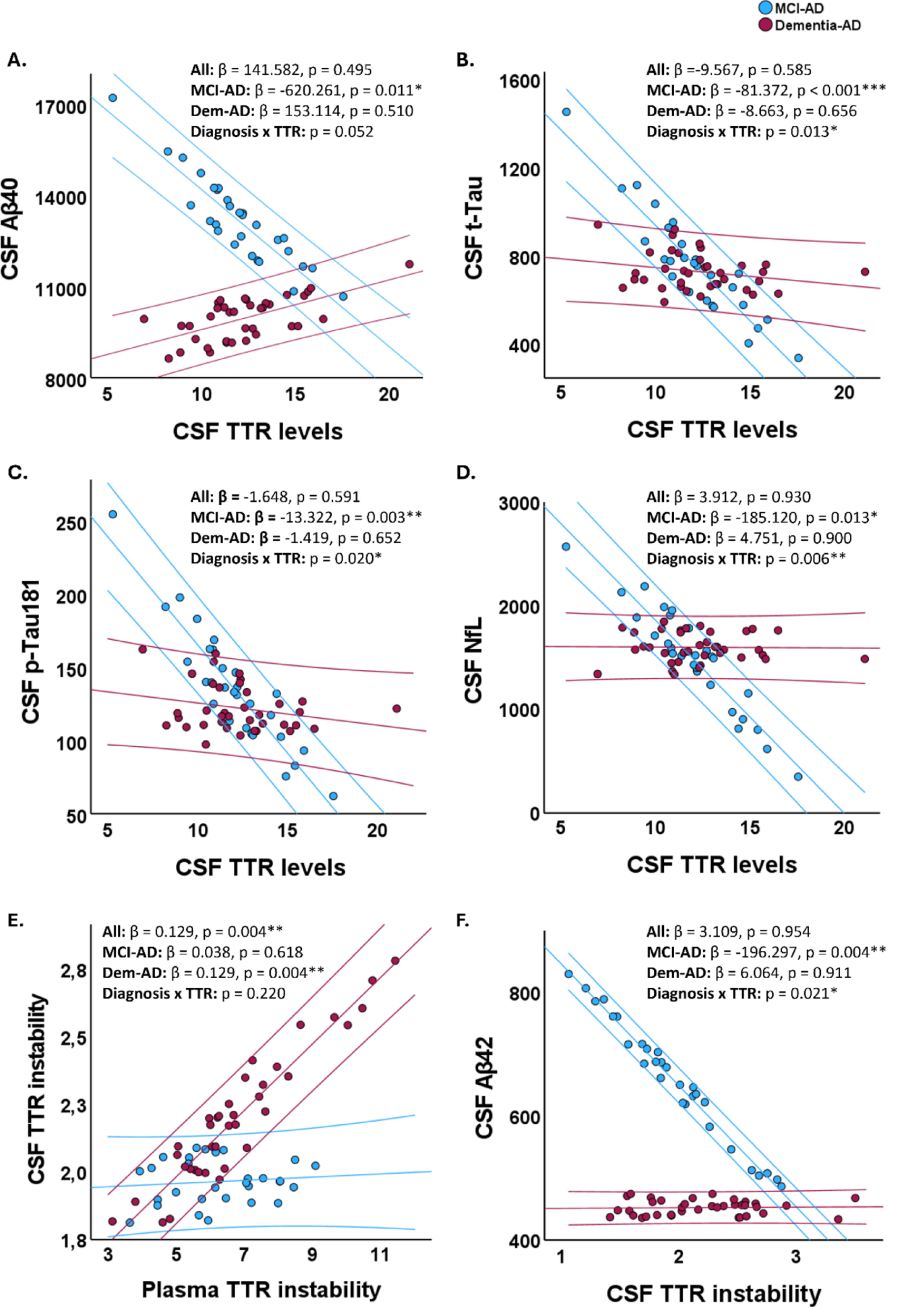
Association between plasma or CSF TTR and its instability or levels with core AD biomarkers in AD. Scatterplots representing the association between TTR and CSF AD-related biomarkers according to clinical stage (MCI-AD vs. Dementia-AD). Associations between CSF TTR levels and (**A**) Aβ40, (**B**) t-Tau, (**C**) p-Tau181, and (**D**) NfL levels. (**E**) Association between plasma and CSF TTR instability. (**F**) Association between CSF TTR instability and CSF Aβ42 levels. Each point represents an individual participant. The central line represents the adjusted regression line for each diagnostic group (blue, MCI-AD; red, Dementia-AD), while the outer lines indicate the 95% confidence intervals of the fitted models. Standardized regression coefficients (β) and p values are displayed for the stratified analyses and were computed using linear models adjusted for age and sex. In addition, a Diagnosis x TTR interaction term was included in the models to assess whether associations differed across disease stages. All regression lines correspond to models adjusted for age and sex. Asterisks indicate significant differences, where *p < 0.05,**p < 0.01 and ***p < 0.001. Abbreviations: Aβ - amyloid-β, AD - Alzheimer’s disease, CSF - cerebrospinal fluid, MCI - mild cognitive impairment, NfL - neurofilament light chain, p-Tau - phosphorylated Tau, t-Tau- total Tau.

Across the whole cohort, no main effects of CSF TTR levels were observed on CSF Aβ40, p-Tau181, t-Tau, or NfL concentrations after covariate adjustment. However, the CSF TTR × diagnosis interaction term reached significance for t-Tau (*p* = 0.013), p-Tau181 (*p* = 0.020), and NfL (*p* = 0.006), and showed a trend toward significance for Aβ40 (*p* = 0.052), indicating that the strength and direction of associations differ across diagnostic stages (Fig. 2A-D).

In stratified analyses, lower CSF TTR levels were significantly associated with higher CSF Aβ40 (β = −620.3, *p* = 0.011), t-Tau (β = −81.4, *p* < 0.001), p-Tau181 (β = −13.3, *p* = 0.003), and NfL (β = −185.1, *p* = 0.013) among MCI-AD participants (Fig. 2A-D). These relationships were not evident in the Dementia-AD group.

CSF and plasma TTR instability were significantly associated in the whole cohort (β = 0.129, *p* = 0.004, Fig. 2E). Notably, higher CSF TTR instability was linked to lower CSF Aβ42 levels in MCI-AD (β = −196.3, *p* = 0.004), while this relationship was absent in Dementia-AD (Fig. 2F). All other tested associations were non-significant after adjustment.

Additional exploratory correlations between TTR levels or instability and demographic or clinical variables were performed using Spearman’s correlation test and are presented in Supplementary Fig. S1.

### Influence of the APOE ε4 genotype on TTR levels and stability

**Fig. 3:**
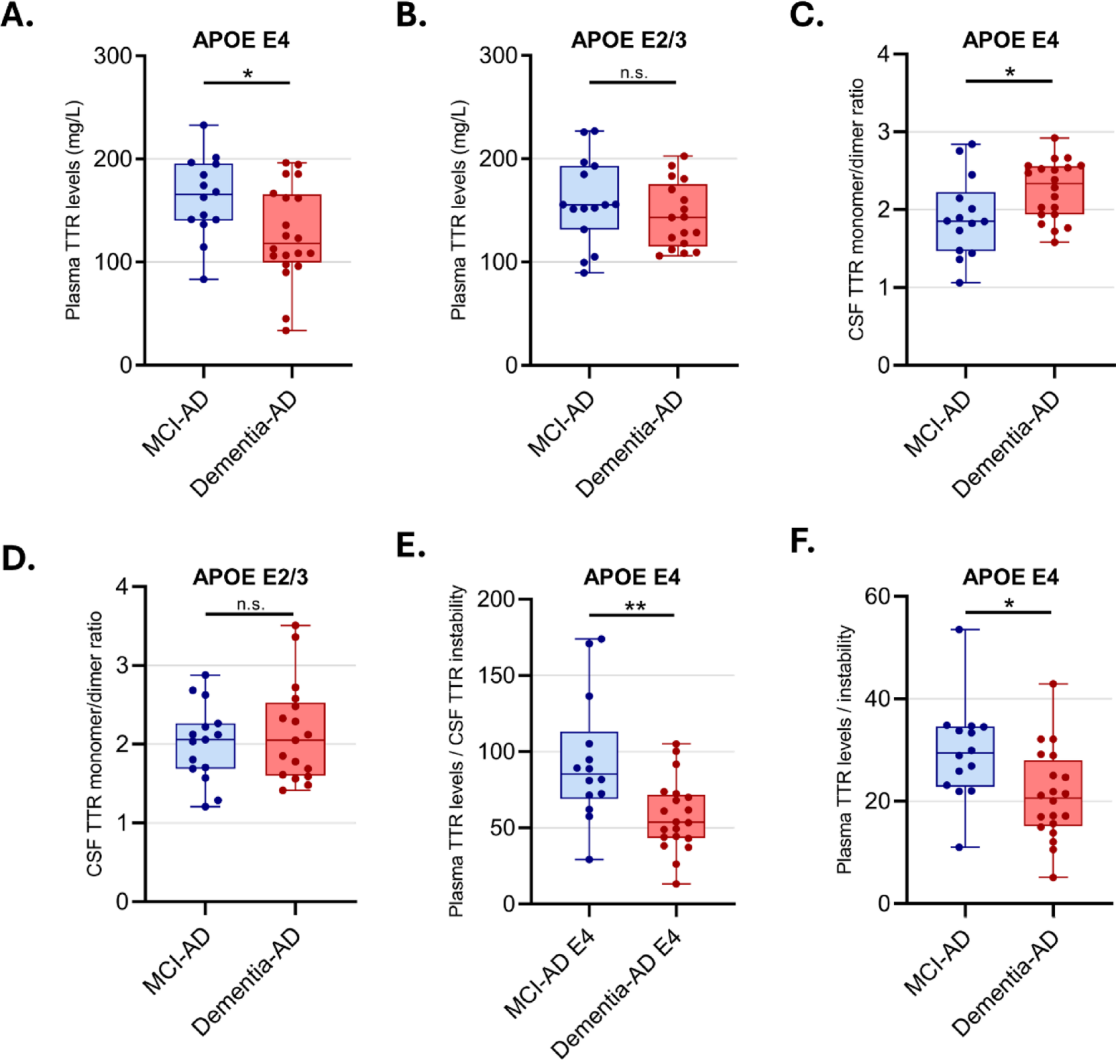
Influence of APOE genotype on TTR levels and instability. Box plots comparing plasma TTR levels and CSF TTR tetrameric instability between MCI-AD and Dementia-AD patients stratified by APOE genotype. Plasma TTR levels in ε4 carriers (**A**) and ε2/ε3 carriers (**B**). CSF TTR instability in ε4 carriers (**C**) and ε2/ε3 carriers (**D**), expressed as the ratio of monomer/dimer.(**E**) Ratio TTR Plasma levels/CSF instability and (**F**) ratio TTR Plasma levels/instability in ε4 carriers. Asterisks indicate significant differences, where * *p* < 0.05 and ** *p* < 0.01, by two-tailed Mann-Whitney test.

Next, we examined whether APOE genotype modulates TTR levels and/or stability. We categorized patients into two groups based on APOE status: APOE ε2/ε3 (no ε4 allele) and APOE ε4 carriers. Within each genotype group, MCI-AD and Dementia-AD patients were compared for plasma TTR levels, CSF TTR levels, and its respective instability. In APOE ε4 carriers, plasma TTR levels were notably lower in Dementia-AD (Fig. 3A). Although plasma TTR levels showed a slight decrease in Dementia-AD compared to MCI-AD in the APOE ε2/ε3 genotype, this difference did not reach significance (Fig. 3B).

Also, in ε4 genotype, CSF TTR instability was increased in Dementia-AD (Fig. 3C), but not ε2/ε3 genotype (Fig. 3D), suggesting that APOE ε4 carriers might be more prone to TTR destabilization in the CNS. CSF TTR levels and plasma TTR instability showed no significant changes between MCI-AD and Dementia-AD in both genotypes (data not shown).

Furthermore, the ratios of plasma TTR levels to CSF instability or plasma instability also allowed discrimination between MCI-AD and Dementia-AD groups within the APOE ε4 genotype (Fig. 3E, F).

### Evaluating TTR tetramer destabilization by Aβ

**Fig. 4:**
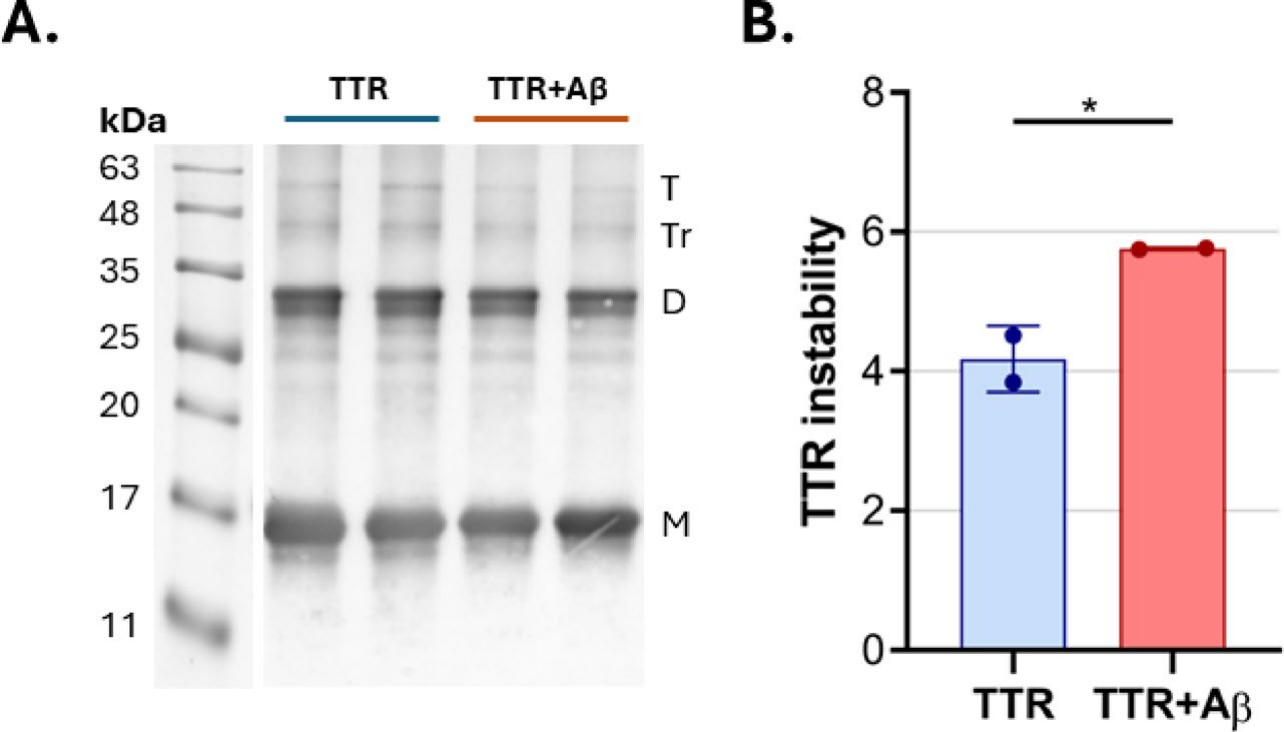
Effect of TTR binding to Aβ42 peptide and consequences in TTR tetrameric instability. (**A**)Western blot analysis of TTR (10 μM) alone or in combination with Aβ42 (40 μM) after incubation at 37 °C for 24 hours, detected using an anti-TTR antibody. Samples were subjected to semi-denaturing gel electrophoresis. The positions of TTR monomer (M), dimer (D), trimer (Tr), and tetramer (T) are indicated on the right side of the blot, with molecular weights in kDa marked on the left. (**B**) Graphical analysis quantifying TTR instability, represented by the monomer+dimer/tetramer intensity ratio. Data are presented as mean ± SD. Asterisk indicates significant differences, where * p < 0.05 by unpaired Student’s t test.

To address the question of whether or not TTR instability can be influenced by Aβ peptide, recombinant TTR (10 µM) was incubated with or without Aβ42 (40 µM) for 24 h. TTR instability was assessed using semi-denaturing gel electrophoresis, followed by Western blot analysis. Results showed a clear decrease in TTR instability in samples incubated with Aβ42 for 24 h (Fig. 4).

## Discussion

TTR is known to play an important role in the modulation of Aβ in AD. Experimental studies have shown that TTR can bind Aβ peptides, preventing their aggregation^21–26^ and facilitating clearance across the BBB^30^. Nevertheless, data on TTR levels and particularly on its tetrameric instability in human AD cohorts remain limited^37^, and the association between these parameters and core AD biomarkers is still not fully understood. In this context, our study aimed to investigate how TTR levels and tetrameric stability in plasma and CSF relate to Aβ pathology, neurodegeneration, and disease stage.

We found significantly reduced plasma TTR levels in Dementia-AD patients compared to MCI-AD subjects, consistent with earlier studies^66,67^. Our result suggests a progressive decrease in TTR levels along the AD continuum, as described in several reports comparing AD and control groups^32–34,66,68^. Notably, our study is the first to report sex-specific differences within the AD continuum, where female Dementia-AD subjects exhibited lower TTR levels compared to their MCI-AD counterparts. Previous studies have detected significant decreases in TTR levels among women with AD compared to controls, although differences were not found between MCI and AD. Reduced estradiol, known to regulate hepatic and choroid-plexus TTR expression^69–71^, may partly explain these differences. Experimental models confirm that estradiol enhances TTR expression in the brain and reduces Aβ burden^72^, supporting the hypothesis that women are at a higher risk of developing AD. Although plasma TTR levels declined with disease severity, CSF levels remained relatively stable. CSF TTR levels have been reported to be lower in AD compared to MCI patients^73,74^. However, results across studies have been inconsistent, with some reporting lower CSF TTR levels in AD compared to controls^74–77^, and others finding no difference or even increased levels^78–80^.

TTR is a homotetrameric protein whose structural stability is essential for its physiological and neuroprotective functions. Destabilization of the tetramer reduces its capacity to bind Aβ peptides and may compromise its role in Aβ clearance and brain homeostasis^34,36,37^. In our study, TTR instability, evaluated by the monomer/dimer ratio, was used as a surrogate marker for impaired function. Although we did not detect significant group differences in instability, a trend toward higher CSF TTR instability in Dementia-AD supports the hypothesis of progressive disruption of TTR homeostasis.

To better understand the complex dynamics of TTR in relation to AD pathology, we analyzed associations between TTR and CSF biomarkers using linear models adjusted for age and sex. The analysis revealed stage-dependent effects, with significant associations restricted to the MCI-AD group. In this early stage, CSF TTR levels were negatively associated with p-Tau181, t-Tau, NfL and Aβ40, indicating that reduced TTR corresponds to greater neuronal injury. Additionally, we also found a positive association between CSF and plasma TTR instabilities, reflecting parallel alterations in CSF and plasma TTR. Moreover, CSF TTR instability was negatively associated with CSF Aβ42 levels, supporting the link between impaired TTR stability and reduced amyloid clearance.

Based on these findings, we further showed that Aβ42 can destabilize recombinant TTR tetramers. This is consistent with previous studies that show that Aβ40 can destabilize the TTR tetramer^81^. It can be speculated that under physiological conditions and possibly in the early stages of AD pathogenesis, CSF TTR may sequester Aβ42 and inhibit amyloid formation. Conversely, impaired TTR tetrameric stability and the resulting decrease in TTR levels, driven by elevated Aβ42 concentrations, leads to further Aβ42 accumulation and, consequently, reduced CSF Aβ42 levels. Previous research showing that CSF TTR levels correlate negatively with disease severity and senile plaque abundance^74,76^, and positively with Aβ38, Aβ40, and Aβ42 CSF levels in AD^77^, are consistent with this model.

Importantly, our stratified analyses reveal a clear stage-dependent pattern in the relationship between TTR and AD pathology. In MCI-AD, CSF TTR levels and instability showed robust associations with Aβ40, Aβ42, t-Tau, p-Tau181 and NfL, indicating that TTR remains responsive to the evolving amyloid-Tau neurodegeneration cascade in this early stage. In contrast, none of these associations were present in Dementia-AD, despite lower plasma TTR levels in this group. The absence of biomarker associations in Dementia-AD suggests that once Tau pathology and neurodegeneration are fully established, CSF TTR no longer reflects brain pathology. Together, these findings indicate that TTR acts as an early-stage biomarker tightly linked to Aβ- and Tau-related mechanisms, while in advanced AD its dynamics are increasingly influenced by other factors.

Further complexity arises from evidence that CSF TTR is not exclusively determined by choroid plexus synthesis. Studies in hereditary ATTR V30M patients who undergo liver transplantation demonstrate that significant WT TTR originating from the periphery appears in the CSF post-transplant, indicating that circulating TTR can enter the CSF compartment^82,83^. This mechanism becomes particularly relevant in advanced AD, where choroid plexus dysfunction and reduced CSF turnover may increase the influence of peripheral TTR on CSF homeostasis^84^. In line with this, the only significant association observed in our Dementia-AD group was between plasma TTR and CSF TTR instability, suggesting a greater relative contribution of peripheral TTR in later disease stage.

This peripheral influence becomes particularly relevant when considering that, although CSF TTR showed pathological associations, the most pronounced alteration occurred in plasma TTR levels. This decrease in peripheral TTR could be attributed to its regulation in the liver, where TTR synthesis can be modulated independently of the CP. For instance, TTR is downregulated in the liver during the acute phase response to inflammatory conditions, a process that occurs without corresponding transcriptional changes in the CP^85–87^. The implications of this regulation within the context of AD, however, require further investigation to elucidate the impact on disease progression. With aging, TTR becomes more amyloidogenic and susceptible to oxidative and metal-ion stress, promoting tetramer dissociation and clearance. Additionally, TTR deposition has been observed in the cerebral vasculature and plaques of AD patients^88–90^, suggesting that partial aggregation and sequestration could further reduce its circulating levels. These systemic mechanisms may therefore dominate TTR alterations in Dementia-AD.

Our study has several strengths. The cohort reflects routine clinical practice, reflecting real-life patients seeking medical attention in hospital environments. All participants underwent comprehensive diagnostic evaluations with overlapping measures to enhance diagnostic consistency, including analyses of well-validated CSF biomarkers. The availability of paired plasma and CSF samples from the same individuals enabled us to examine TTR levels and tetrameric stability across compartments. Furthermore, the analysis of vascular and metabolic factors, including white matter and basal ganglia hyperintensities, BBB integrity, diabetes, hypertension, and glucose levels, provided a multifaceted view of their impact on disease progression and TTR.

There are also limitations that should be taken into account. This study is limited by its monocentric design, modest cohort size that limits statistical power and absence of age-matched healthy controls. The neurological controls that were available were individuals undergoing routine diagnostic lumbar punctures for conditions such as acute or chronic headaches or peripheral polyneuropathy. Additionally, their average age was significantly lower compared to the patient cohorts. Finally, comorbidities and peripheral inflammation may have influenced TTR regulation. Collectively, these considerations highlight the value of larger and multi-center studies to confirm and extend our observations.

## Conclusions

 Our results indicate that plasma TTR concentration decreases in Dementia-AD, particularly in women, while associations between TTR and key AD markers, such as Aβ pathology and neurodegeneration, are restricted to the MCI-AD stage. These findings highlight the complex and stage-dependent role of TTR in AD progression.

## Supplementary Information

Below is the link to the electronic supplementary material.

Supplementary Material 1

## Data Availability

The datasets used and/or analysed during the current study are available from the corresponding author on reasonable request.

## Declarations

### Preprint

Uploaded to a preprint server (https://www.biorxiv.org/): https://www.biorxiv.org/content/10.1101/2025.01.24.634710v1
