# Supplementary material for: Levels and instability of transthyretin and correlations with core biomarkers in Alzheimer’s disease
Source: Sci Rep. 2026 Mar 12;16:13024. doi: 10.1038/s41598-026-41717-7 (PMC13100035; doi:10.1038/s41598-026-41717-7)

Title: Levels and Instability of Transthyretin and correlations with core biomarkers in Alzheimer's Disease

Tiago Gião[1, 2, 3], *Miguel Tábuas-Pereira*[4,5,6,7], *Inês Baldeiras*[4,5,6], *Joana Saavedra*[1, 2, 3], *Alexandre Dias*[1, 3, 8], *Maria João Saraiva*[1, 2], *Maria Rosário Almeida*[1, 2, 3], *Isabel Santana*[4,5,6,7,*], *Isabel Cardoso*[1, 2, 3, *]

[1]i3S - Instituto de Investigação e Inovação em Saúde, Universidade do Porto, 4200-135, Porto, Portugal.
[2]IBMC - Instituto de Biologia Molecular e Celular, Universidade do Porto, 4200-135, Porto, Portugal.
[3]ICBAS - Instituto de Ciências Biomédicas Abel Salazar, Universidade do Porto, 4050-313, Porto, Portugal.
[4]Faculdade de Medicina, Universidade de Coimbra, 3000-075, Coimbra, Portugal.
[5]CIBB - Centro de Inovação em Biomedicina e Biotecnologia, Universidade de Coimbra, 3004-504, Coimbra, Portugal.
[6]CNC - Centro de Neurociências e Biologia Celular, Universidade de Coimbra, 3004-504, Coimbra, Portugal.
[7]Departmento de Neurologia, Hospital da Universidade de Coimbra, ULS de Coimbra, 3004-561, Coimbra, Portugal.
[8]Ipatimup - Instituto de Patologia e Imunologia Molecular da Universidade do Porto, 4200-135, Porto, Portugal.

* These authors contribute equally to this work
Correspondence to: icardoso@ibmc.up.pt (IC) and 7708@chuc.min-saude.pt (IS)

Table S1. Comorbidities and health status of the cohort.

	**MCI-AD (n = 29)**	**Dementia-AD (n = 37)**	**P**
Diabetes mellitus, n (%)	7 (24.14)	4 (10.81)	0.191
Dyslipidemia, n (%)	14 (48.28)	17 (45.95)	0.851
Obesity, n (%)	4 (13.79)	5 (13.51)	0.194
Hypertension, n (%)	16 (56.67)	22 (59.46)	0.727
Psychiatric issues, n (%)	9 (31.03)	12 (32.43)	1
Plasma glucose (mg/dL) *	104.94 (21.72)	94.46 (18.73)	0.042
CSF glucose (mg/dL) #	69.69 (13.59)	60.61 (5.59)	0.031
Plasma Albumin (mg/mL)	46.31 (4.81)	45.43 (6.87)	0.531
CSF Albumin (mg/mL)	0.095 (0.027)	0.097 (0.032)	0.872
Qalb	2.07 (0.59)	2.18 (0.86)	0.583

Differences between groups were assessed using Mann-Whitney U for continuous variables and chi-square test (for dyslipidemia, hypertension and psychiatric issues) or Fisher's exact test (for diabetes mellitus and obesity). Data are presented as mean (standard deviation) or number of participants (percentage), as appropriate. P values < 0.05 were considered statistically significant.

Note:

* Sample size differs due to incomplete data for the Glycemia parameter: NC, n=20; MCI-AD, n=17; Dementia-AD, n=28.

# Sample size differs due to incomplete data for the CSF glucose parameter: NC, n=20, MCI-AD, n=16; Dementia-AD, n=28.

Abbreviations used: Qalb - CSF/plasma albumin quotient

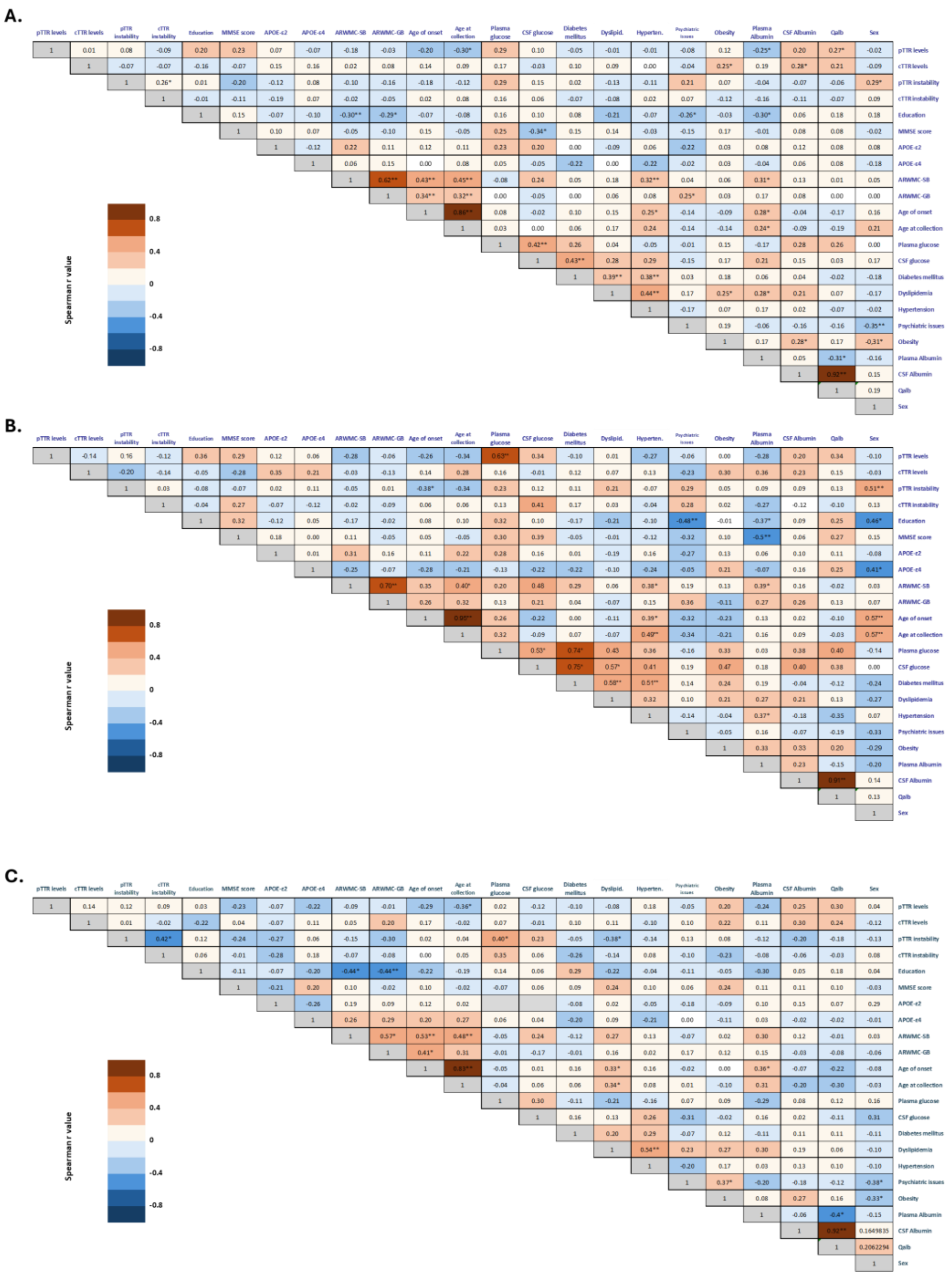

Figure S1. Correlation between plasma (pTTR) and CSF TTR (cTTR) instability and levels with demographic and clinical variables in A. MCI-AD and Dementia-AD (n=66) group; **B.** MCI-AD (n=29) group; **C.** Dementia-AD (n=37) group. Sample size differs due to incomplete data for the plasma glucose parameter (MCI-AD and Dementia-AD, n=45; MCI-AD, n=17; Dementia-AD, n=28) and CSF glucose parameter (MCI-AD and Dementia-AD, n=44; MCI-AD, n=16; Dementia-AD, n=28).

The correlation matrix graphically represents the Spearman's correlation coefficient (r) for each pairwise comparison among the biomarkers. According to the color scale on the right side of the matrix, positive and negative correlations are indicated in shades of red and blue, respectively an r value of +1 indicates a perfect positive relationship, r = 0 indicates no relationship, and an r value of -1 indicates a perfect negative relationship. Significant differences are denoted by * ($p < 0.05$) and ** ($p < 0.01$).

Abbreviations used: APOE - apolipoprotein E, ARWMC - age-related white matter changes, CSF - cerebrospinal fluid, MCI - mild cognitive impairment, MMSE - Mini-Mental State Examination, Qalb - CSF/plasma albumin quotient

Uncropped Western blot images

1. Evaluation of plasma instability

All plasma samples were analyzed in two independent sets of Western blot experiments, with one replicate per sample. A total of 12 gels were run for each set, and the results were visualized in six images, each showing two gels. The arrangement of the samples in each gel is detailed in a corresponding template displayed above the respective blot. For each gel, a molecular weight marker (MW marker) and recombinant TTR (recTTR) were included as controls. Each gel contains eight plasma samples from patients with Mild Cognitive Impairment-AD (MCI-AD) or AD Dementia (Dem.-AD). Some blots also include non-demented Control (NC) samples, which were not analyzed within the scope of this study. Monomers and dimers are represented in the images, and the ratio of the band intensities of monomers to dimers was used to evaluate plasma instability. All images were acquired with a 1 second exposure time. The general scheme of the blots is as follow:

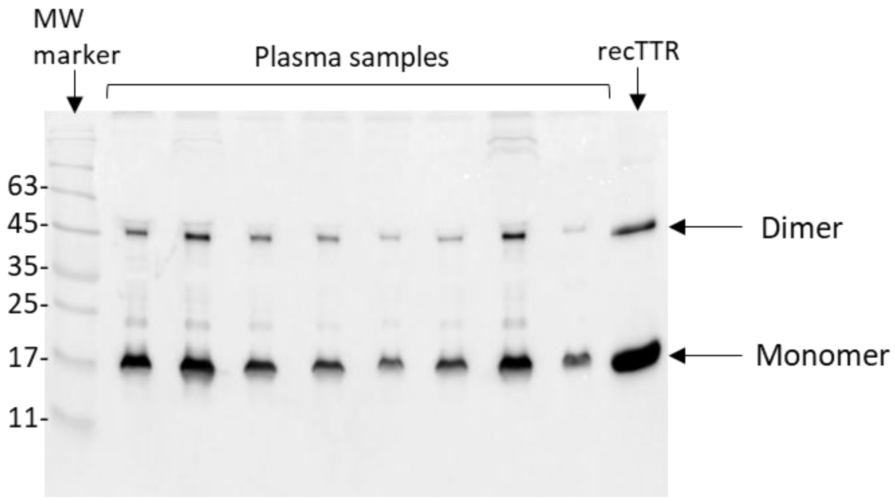

1.1. Evaluation of plasma instability – 1st set of blots (12 blots)

Blot 1 and 2

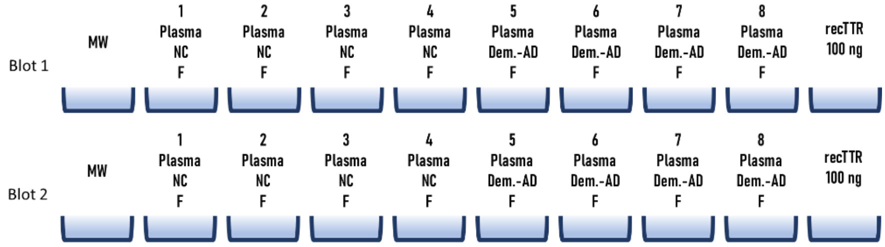

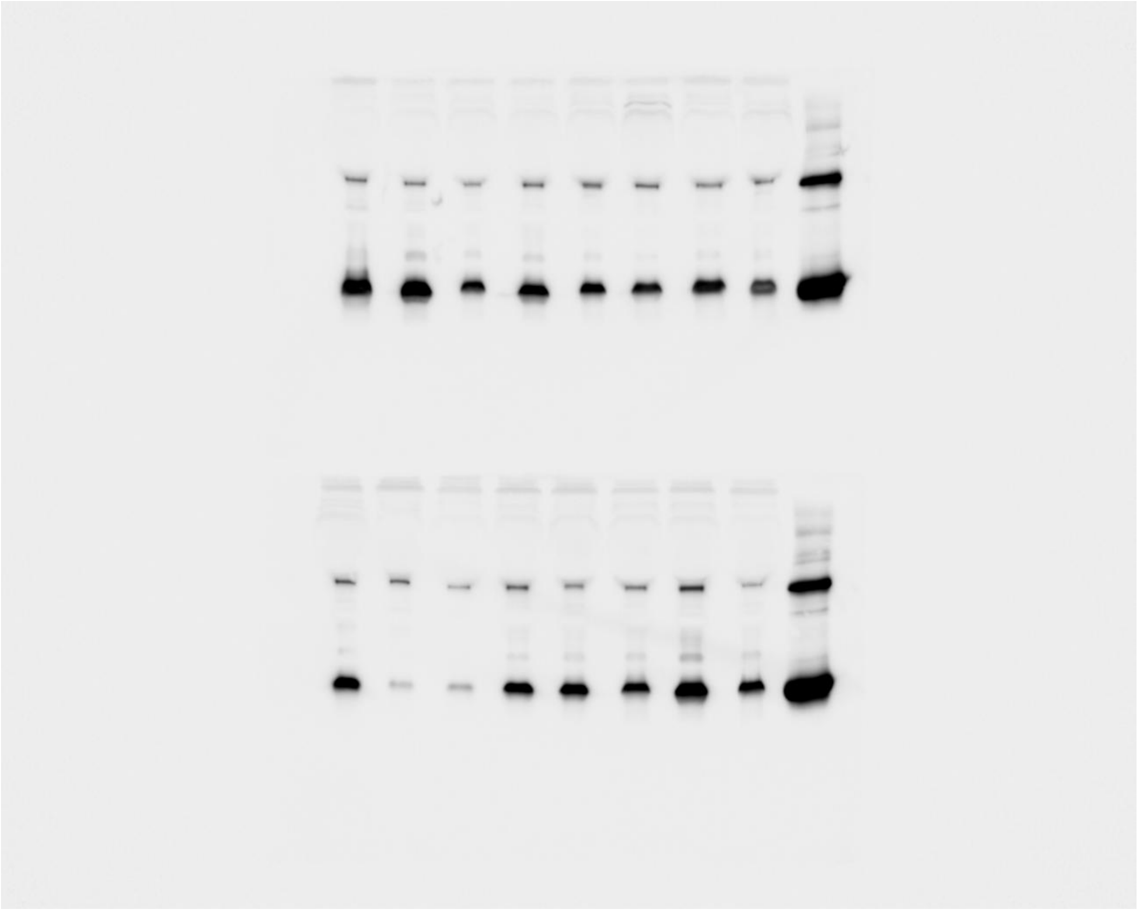

Blot 3 and 4

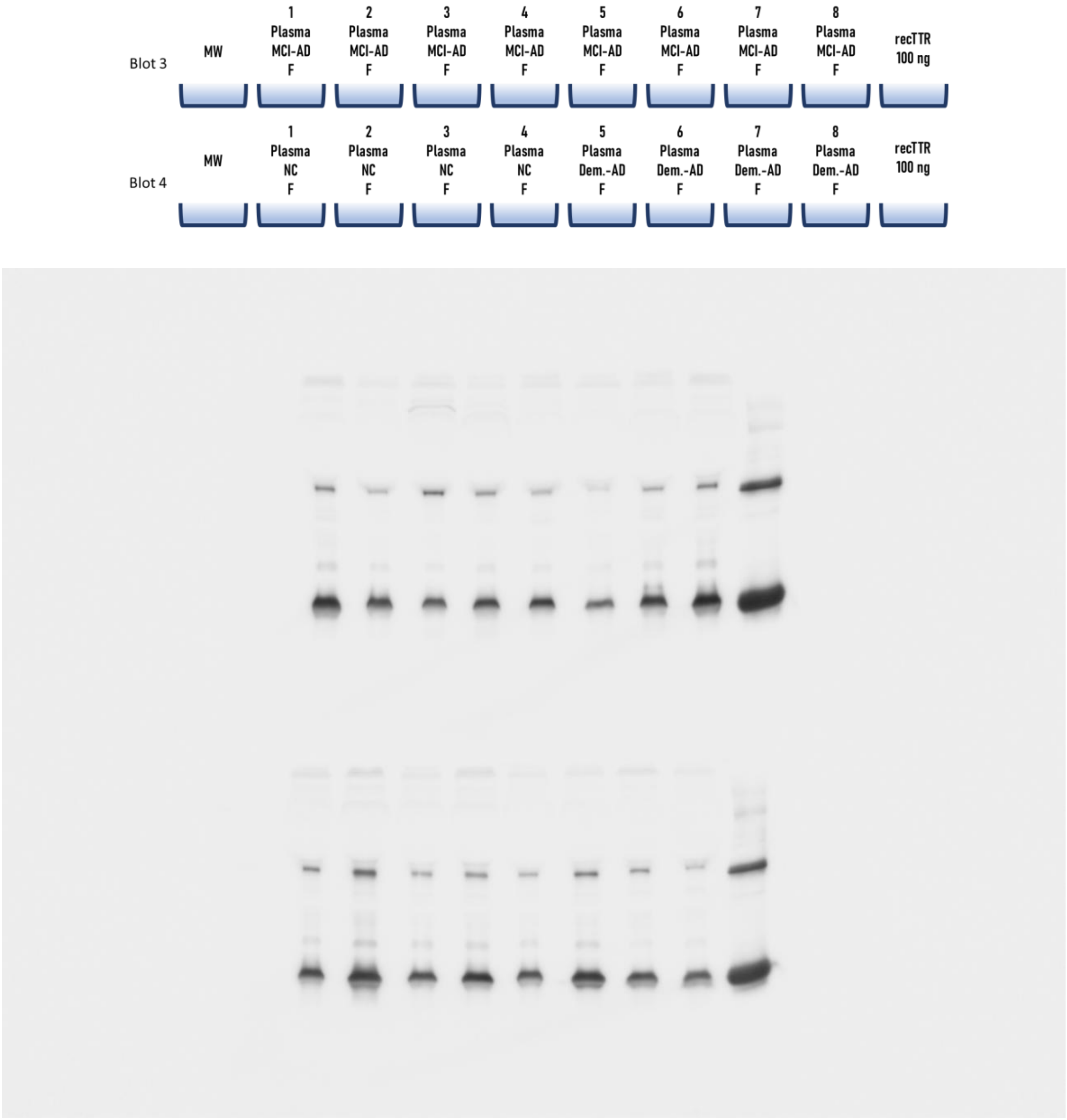
Blot 3
MW
1 Plasma MCI-AD F
2 Plasma MCI-AD F
3 Plasma MCI-AD F
4 Plasma MCI-AD F
5 Plasma MCI-AD F
6 Plasma MCI-AD F
7 Plasma MCI-AD F
8 Plasma MCI-AD F
recTTR 100 ng
Blot 4
MW
1 Plasma NC F
2 Plasma NC F
3 Plasma NC F
4 Plasma NC F
5 Plasma Dem.-AD F
6 Plasma Dem.-AD F
7 Plasma Dem.-AD F
8 Plasma Dem.-AD F
recTTR 100 ng

Blot 5 and 6

Blot 5	MW	1 Plasma NC M	2 Plasma NC M	3 Plasma NC M	4 Plasma NC M	5 Plasma Dem.-AD M	6 Plasma Dem.-AD M	7 Plasma Dem.-AD M	8 Plasma Dem.-AD M	recTTR 100 ng
Blot 6	MW	1 Plasma MCI-AD M	2 Plasma MCI-AD M	3 Plasma MCI-AD M	4 Plasma MCI-AD M	5 Plasma MCI-AD M	6 Plasma Dem.-AD M	7 Plasma Dem.-AD M	8 Plasma Dem.-AD M	recTTR 100 ng

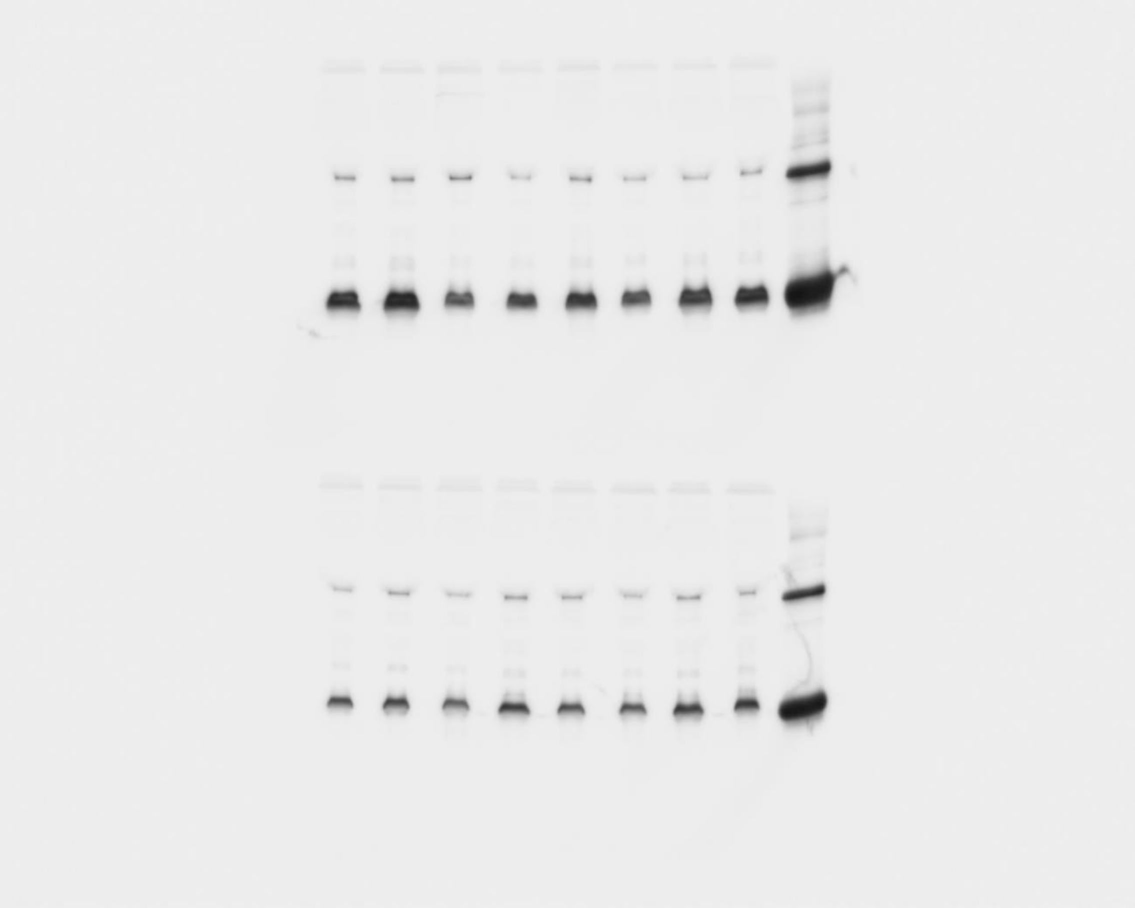

Blot 7 and 8

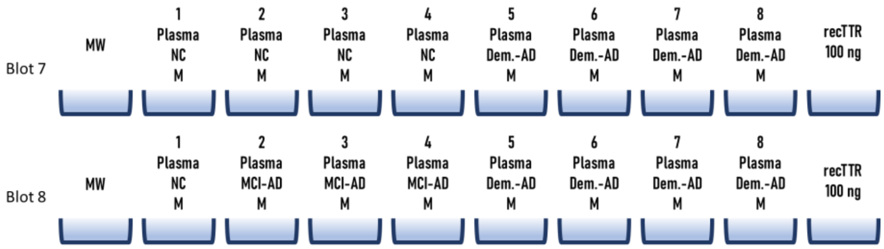

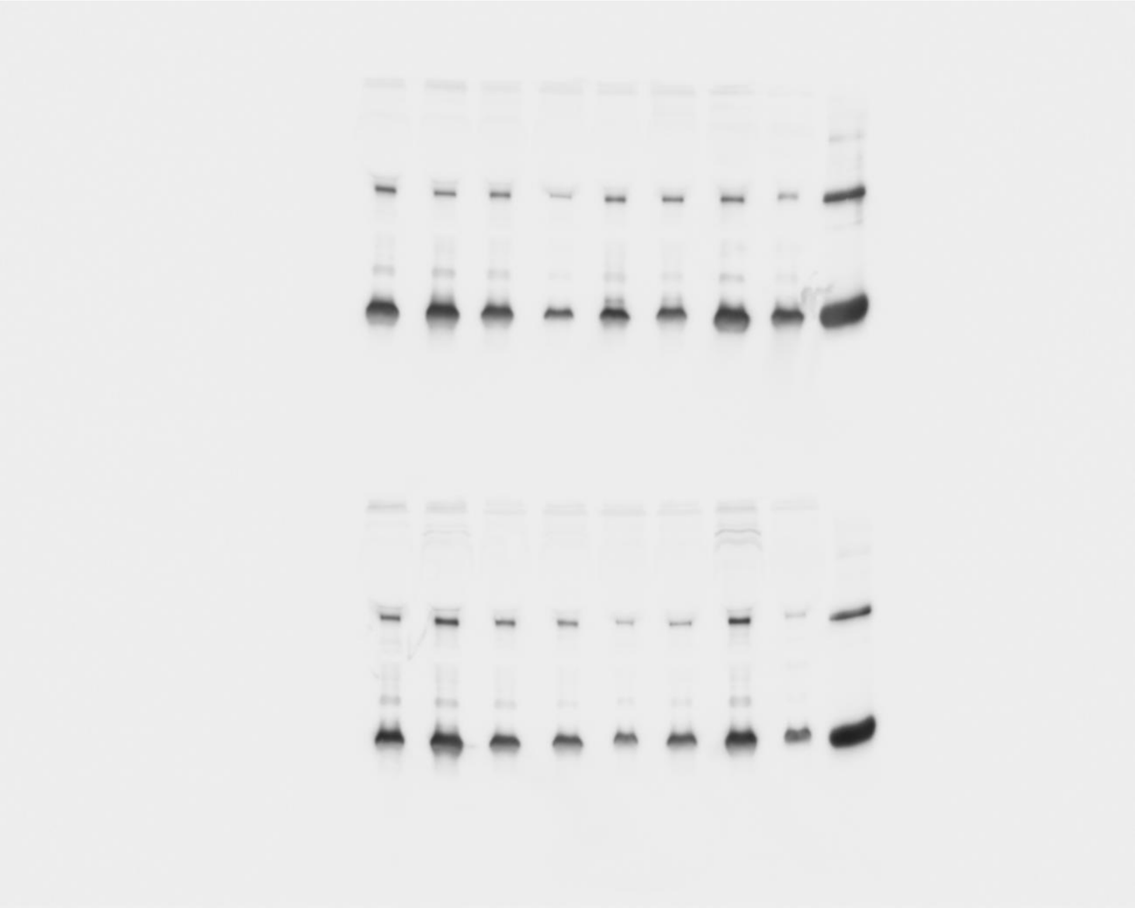

The bottom part of the image shows the uncropped blot presented in Figure 1H.

Blot 9 and 10

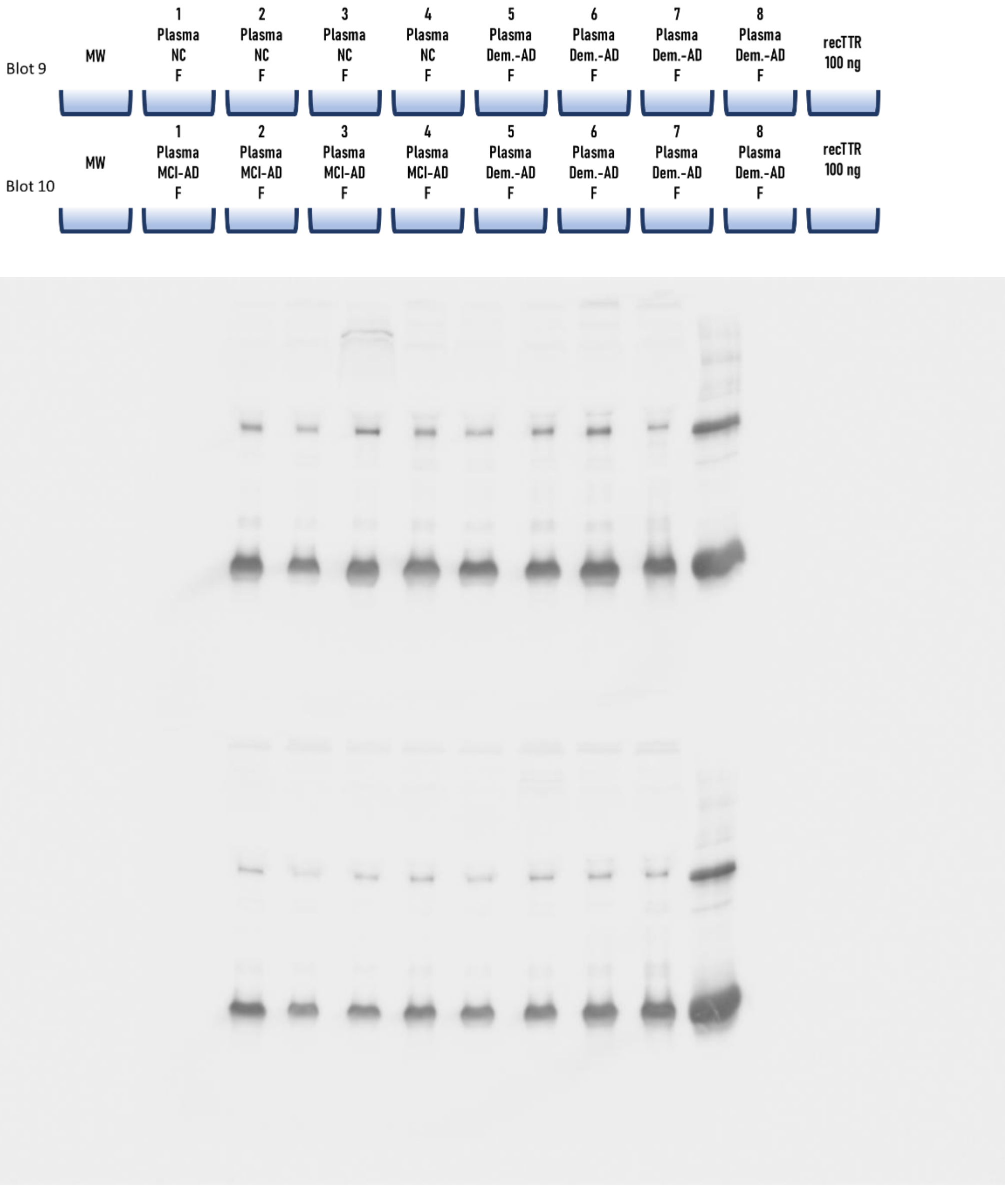
Blot 9
MW
1 Plasma NC F
2 Plasma NC F
3 Plasma NC F
4 Plasma NC F
5 Plasma Dem.-AD F
6 Plasma Dem.-AD F
7 Plasma Dem.-AD F
8 Plasma Dem.-AD F
recTTR 100 ng
Blot 10
MW
1 Plasma MCI-AD F
2 Plasma MCI-AD F
3 Plasma MCI-AD F
4 Plasma MCI-AD F
5 Plasma Dem.-AD F
6 Plasma Dem.-AD F
7 Plasma Dem.-AD F
8 Plasma Dem.-AD F
recTTR 100 ng

Blot 11 and 12

Blot 11	MW	1 Plasma NC F	2 Plasma NC F	3 Plasma MCI-AD F	4 Plasma MCI-AD F	5 Plasma MCI-AD F	6 Plasma MCI-AD F	7 Plasma MCI-AD F	8 Plasma MCI-AD F	recTTR 100 ng
Blot 12	MW	1 Plasma MCI-AD M	2 Plasma MCI-AD M	3 Plasma MCI-AD M	4 Plasma MCI-AD F	5 Plasma MCI-AD F	6 Plasma Dem.-AD F	7 Plasma Dem.-AD F	8 Plasma Dem.-AD F	recTTR 100 ng

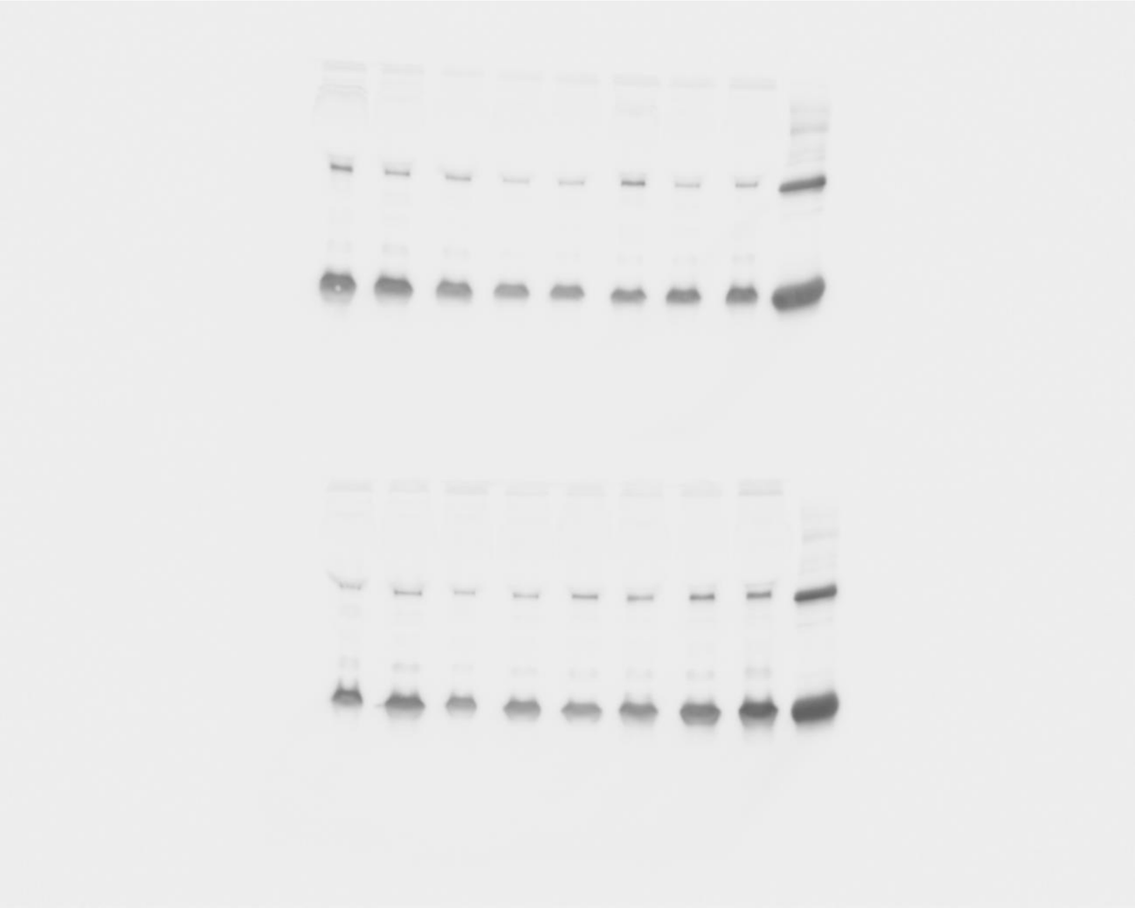

1.2. Evaluation plasma instability – 2nd set of blots (12 blots)

Blot 1 and 2

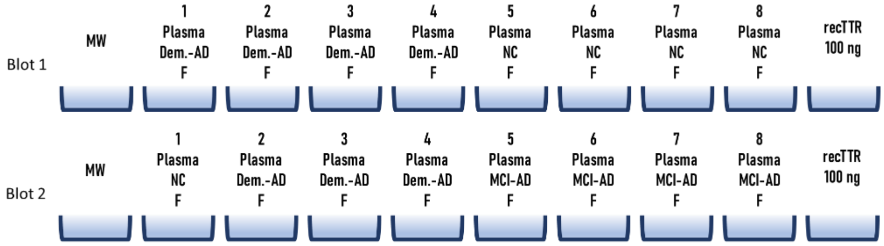

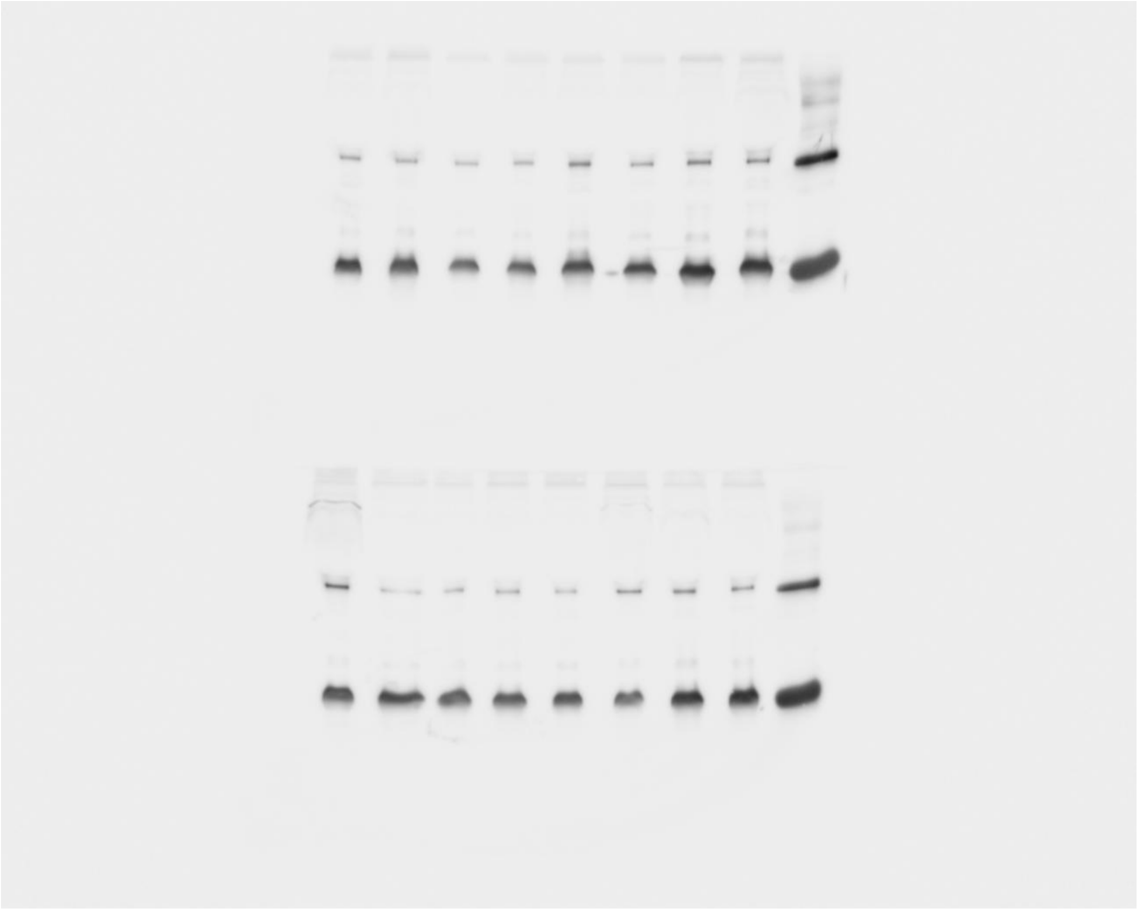

Blot 3 and 4

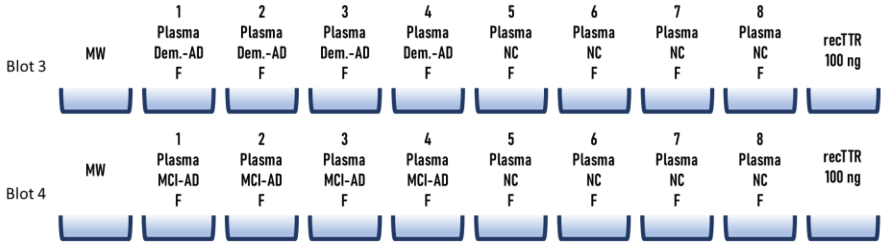
Blot 3
MW
1 Plasma Dem.-AD F
2 Plasma Dem.-AD F
3 Plasma Dem.-AD F
4 Plasma Dem.-AD F
5 Plasma NC F
6 Plasma NC F
7 Plasma NC F
8 Plasma NC F
recTTR 100 ng
Blot 4
MW
1 Plasma MCI-AD F
2 Plasma MCI-AD F
3 Plasma MCI-AD F
4 Plasma MCI-AD F
5 Plasma NC F
6 Plasma NC F
7 Plasma NC F
8 Plasma NC F
recTTR 100 ng

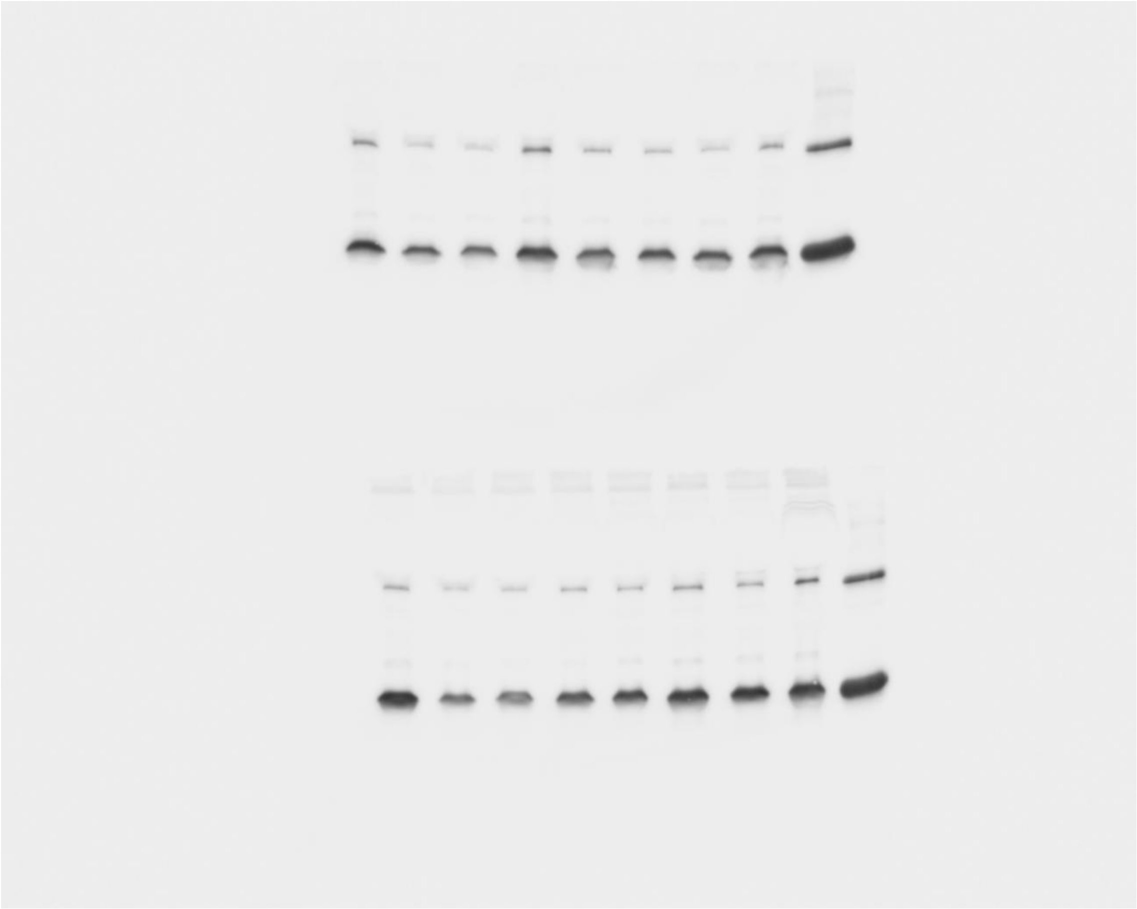

Blot 5 and 6

Blot 5	MW	1 Plasma MCI-AD F	2 Plasma MCI-AD F	3 Plasma MCI-AD F	4 Plasma MCI-AD F	5 Plasma NC F	6 Plasma NC F	7 Plasma NC F	8 Plasma NC F	recTTR 100 ng
Blot 6	MW	1 Plasma Dem.-AD F	2 Plasma Dem.-AD F	3 Plasma Dem.-AD F	4 Plasma Dem.-AD F	5 Plasma MCI-AD F	6 Plasma MCI-AD F	7 Plasma MCI-AD F	8 Plasma MCI-AD F	recTTR 100 ng

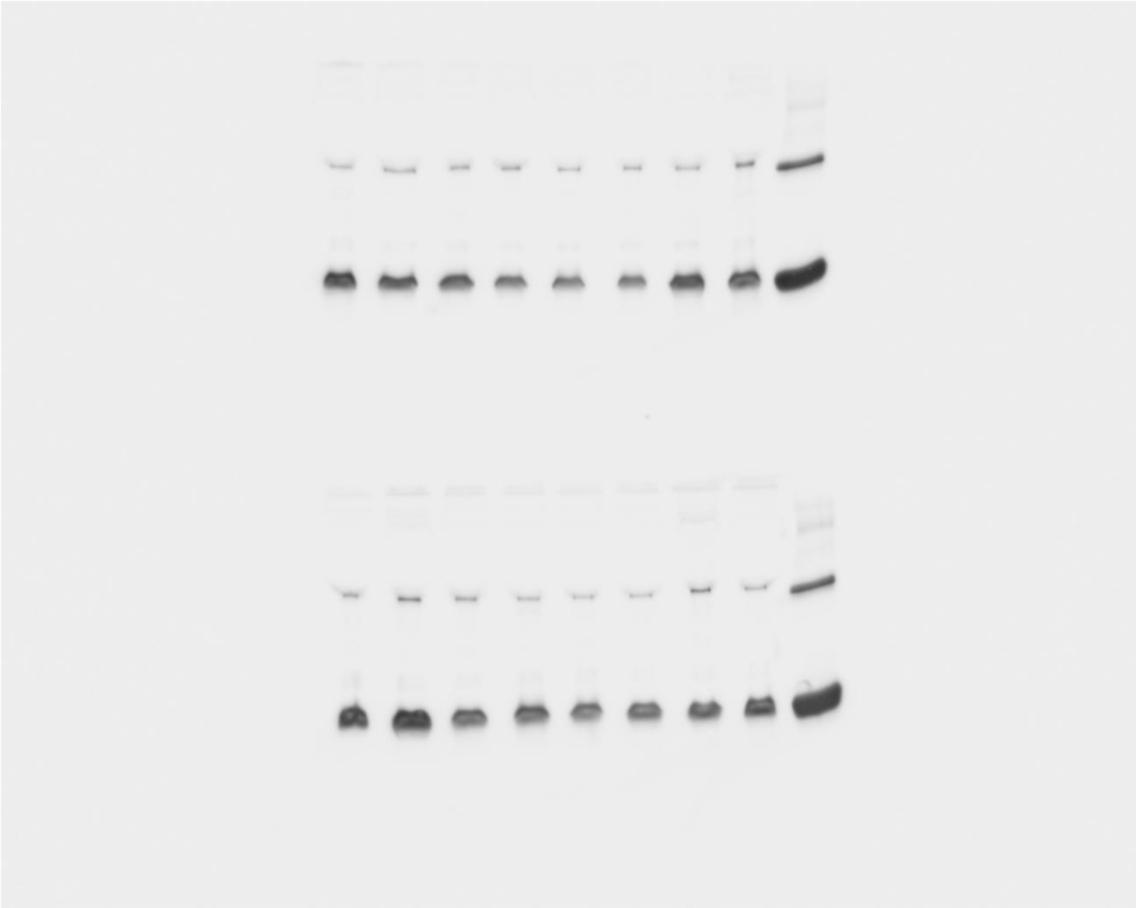

Blot 7 and 8

Blot 7	MW	1 Plasma Dem.-AD F	2 Plasma Dem.-AD F	3 Plasma Dem.-AD F	4 Plasma Dem.-AD F	5 Plasma MCI-AD F	6 Plasma MCI-AD F	7 Plasma MCI-AD F	8 Plasma MCI-AD F	recTTR 100 ng
Blot 8	MW	1 Plasma Dem.-AD F	2 Plasma Dem.-AD F	3 Plasma Dem.-AD F	4 Plasma Dem.-AD F	5 Plasma NC F	6 Plasma Dem.-AD M	7 Plasma Dem.-AD M	8 Plasma MCI-AD M	recTTR 100 ng

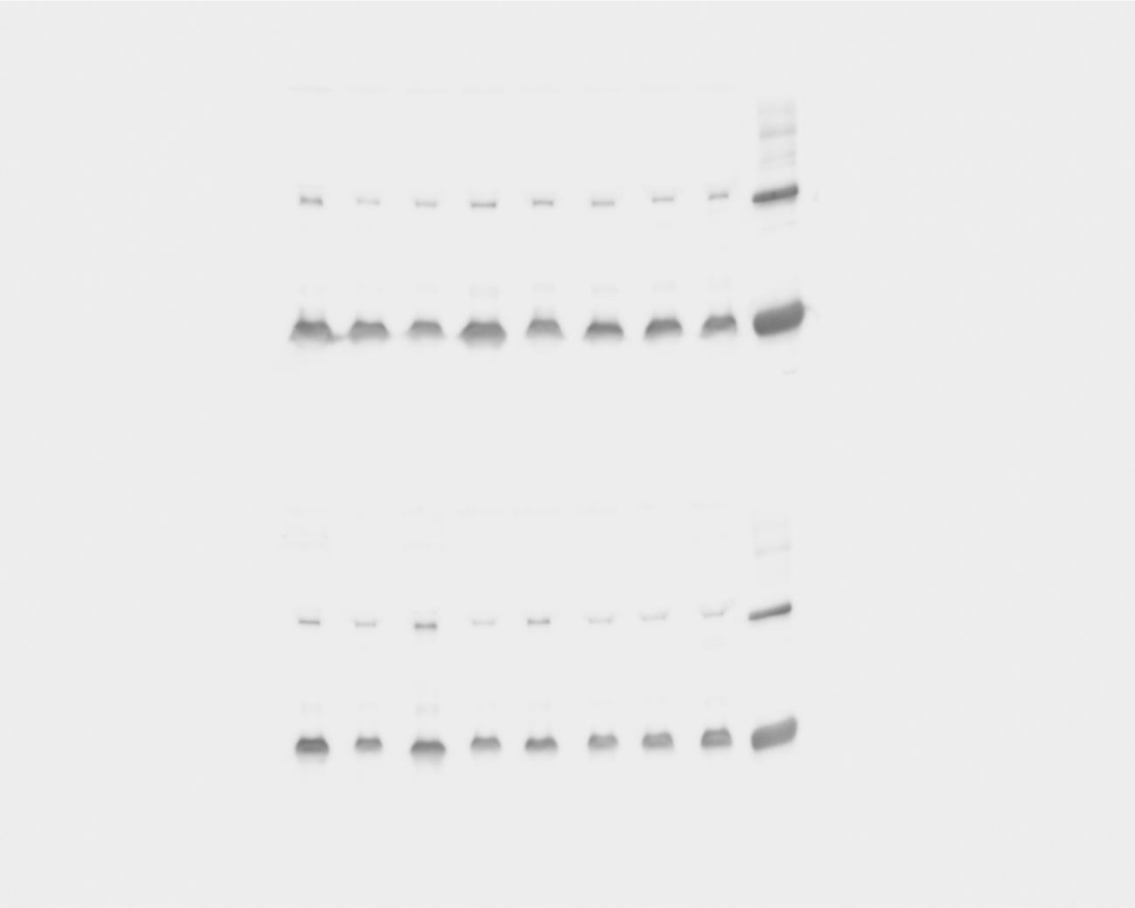

Blot 9 and 10

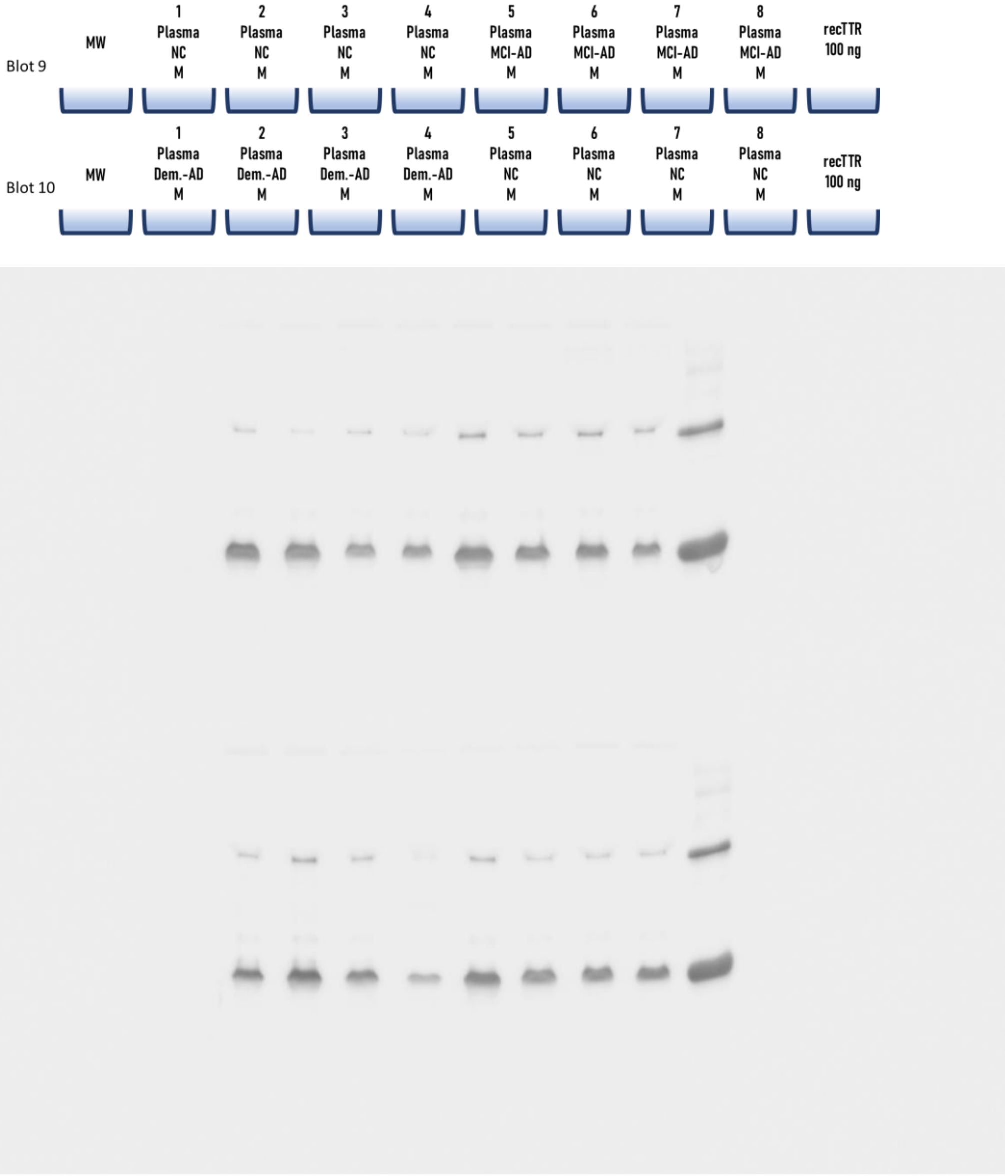
Blot 9
MW
1 Plasma NC M
2 Plasma NC M
3 Plasma NC M
4 Plasma NC M
5 Plasma MCI-AD M
6 Plasma MCI-AD M
7 Plasma MCI-AD M
8 Plasma MCI-AD M
recTTR 100 ng
Blot 10
MW
1 Plasma Dem.-AD M
2 Plasma Dem.-AD M
3 Plasma Dem.-AD M
4 Plasma Dem.-AD M
5 Plasma NC M
6 Plasma NC M
7 Plasma NC M
8 Plasma NC M
recTTR 100 ng

Blot 11 and 12

Blot 11	MW	1 Plasma Dem.-AD M	2 Plasma Dem.-AD M	3 Plasma Dem.-AD M	4 Plasma Dem.-AD M	5 Plasma MCI-AD M	6 Plasma MCI-AD M	7 Plasma MCI-AD M	8 Plasma MCI-AD M	recTTR 100 ng
Blot 12	MW	1 Plasma Dem.-AD M	2 Plasma Dem.-AD M	3 Plasma Dem.-AD M	4 Plasma Dem.-AD M	5 Plasma NC M	6 Plasma MCI-AD M	7 Plasma MCI-AD M	8 Plasma MCI-AD M	recTTR 100 ng

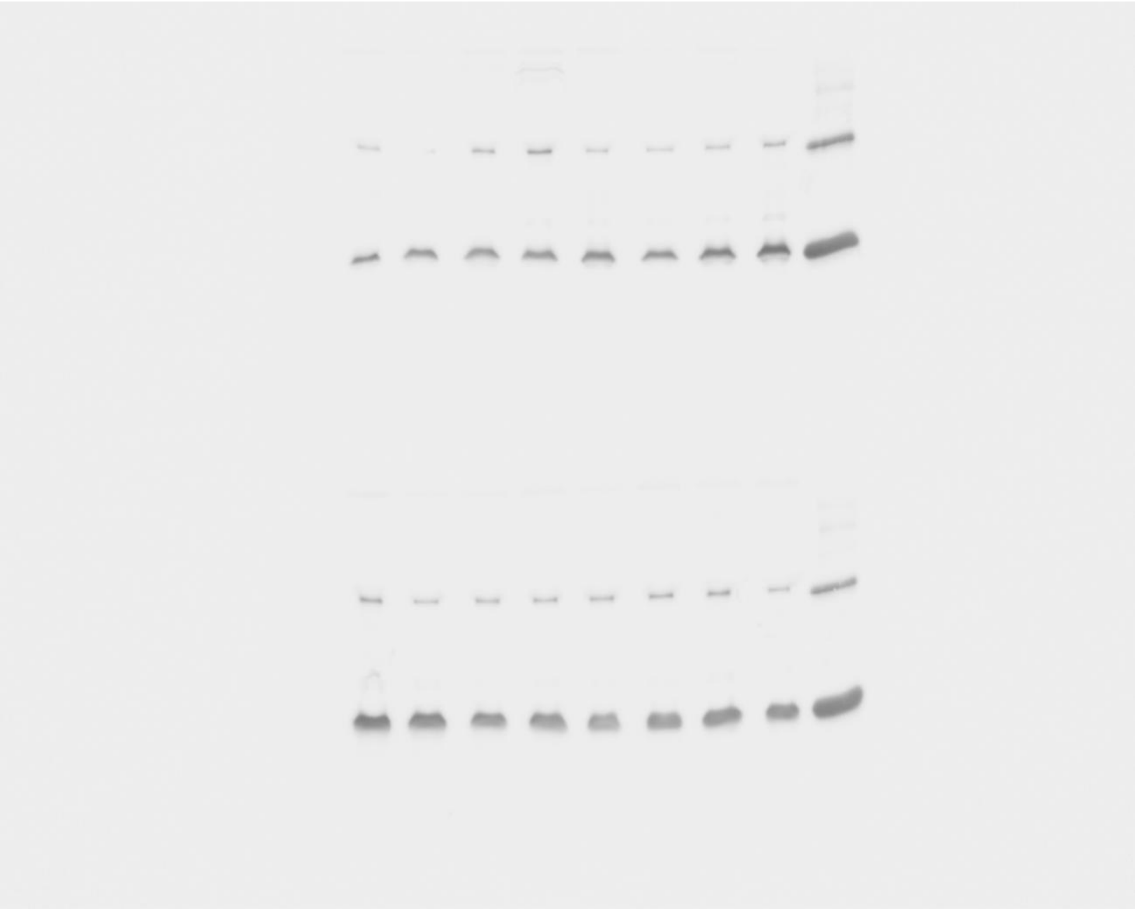

2. Evaluation of CSF instability

All CSF samples were analyzed in two independent sets of Western blot experiments, with one replicate per sample. A total of twelve gels were run for each set, and the results were visualized in six images, each showing two gels. The arrangement of the samples in each gel is detailed in a corresponding template displayed above the respective blot. For each gel, a molecular weight marker (MW marker) and recombinant TTR (recTTR) were included as controls. Each gel contains eight CSF samples from patients with Mild Cognitive Impairment -AD (MCI-AD) or Dementia-AD (Dem.-AD). Some blots also include non-demented control (NC) samples, which were not analyzed within the scope of this study. Monomers and dimers are represented in the images. Monomers were quantified using images acquired with a 1 second exposure time, while dimers were quantified using images acquired with an 11 second exposure time. The ratio of the band intensities of monomers to dimers was used to evaluate CSF instability. The general scheme of the blots is as follow:

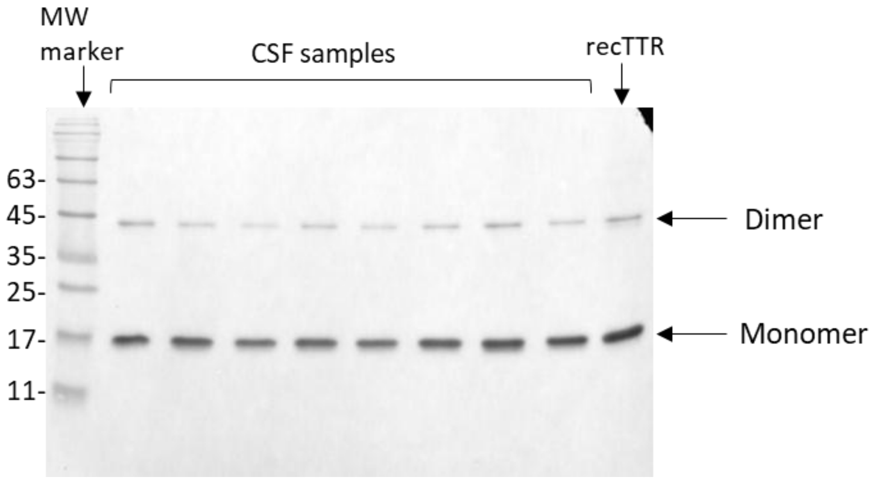

2.1. Evaluation of CSF instability – 1st set of blots (12 blots)

Blot 1 and 2

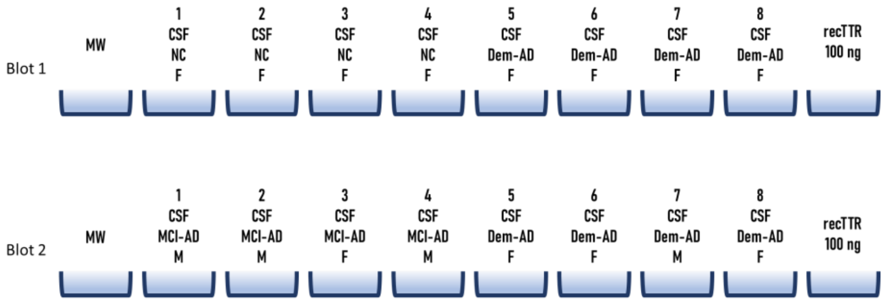

Exposition time = 1 second

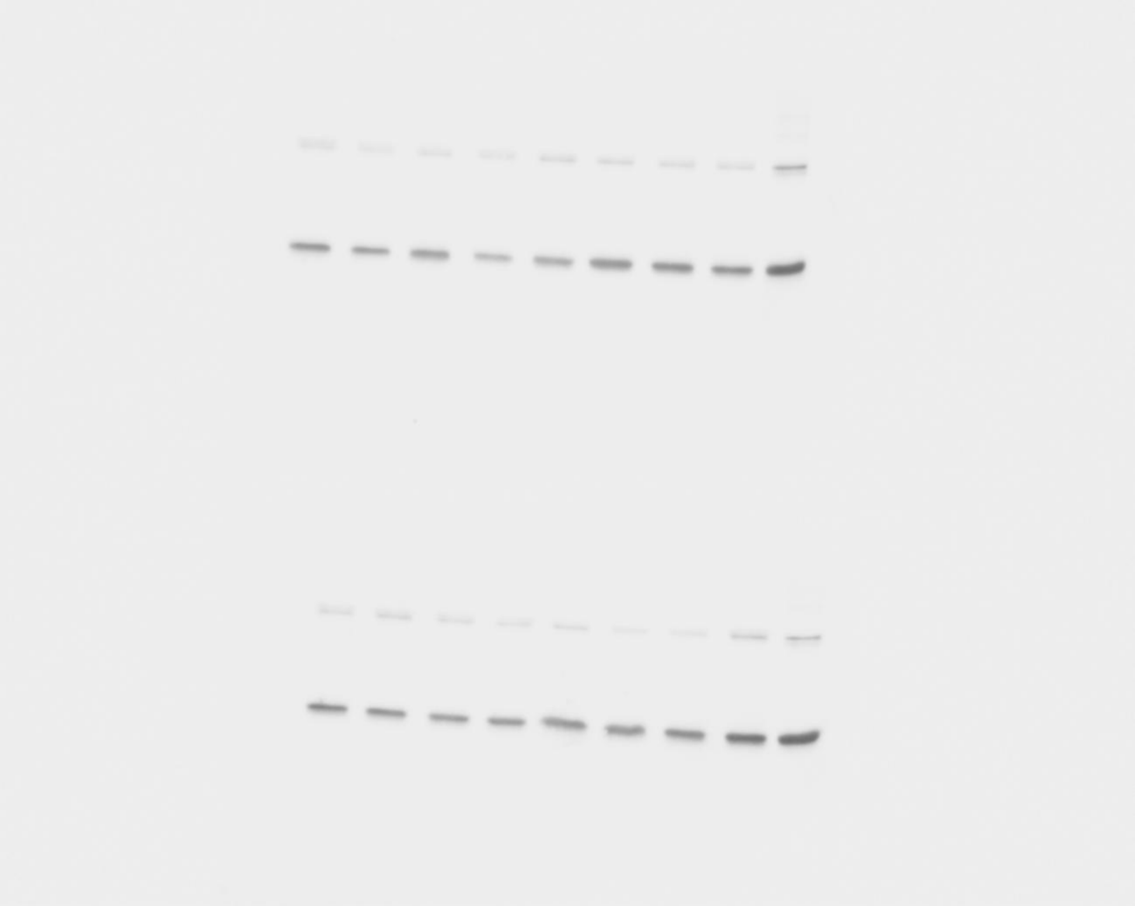

The bottom part of the image shows the uncropped blot presented in Figure 1J.

Exposition time = 11 seconds

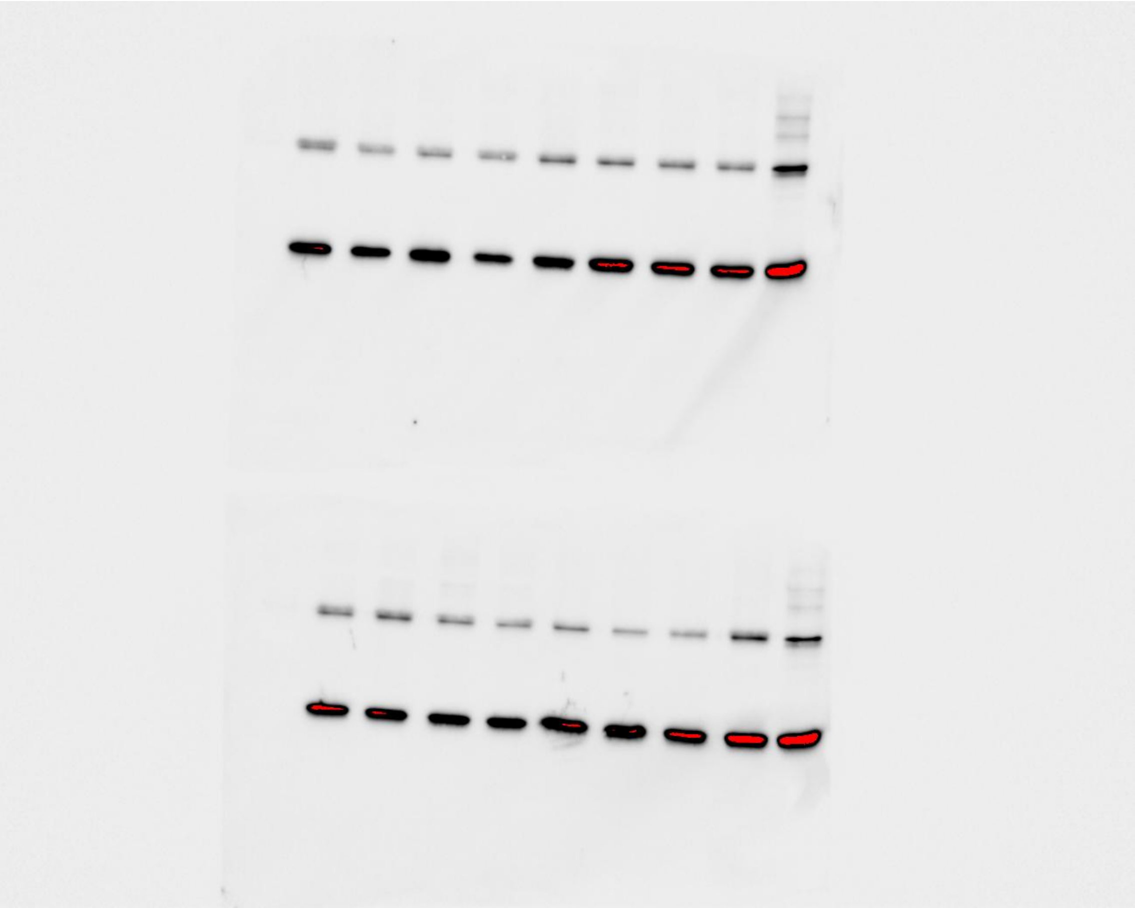

Blot 3 and 4

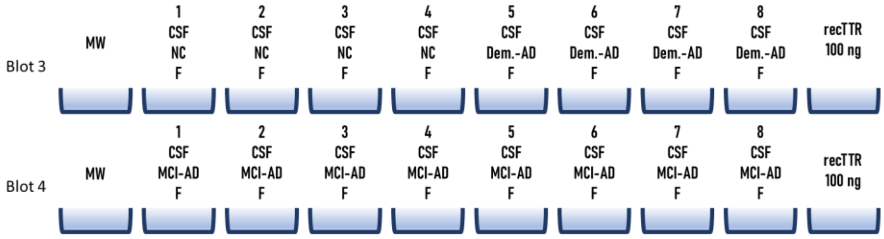

Exposition time = 1 second

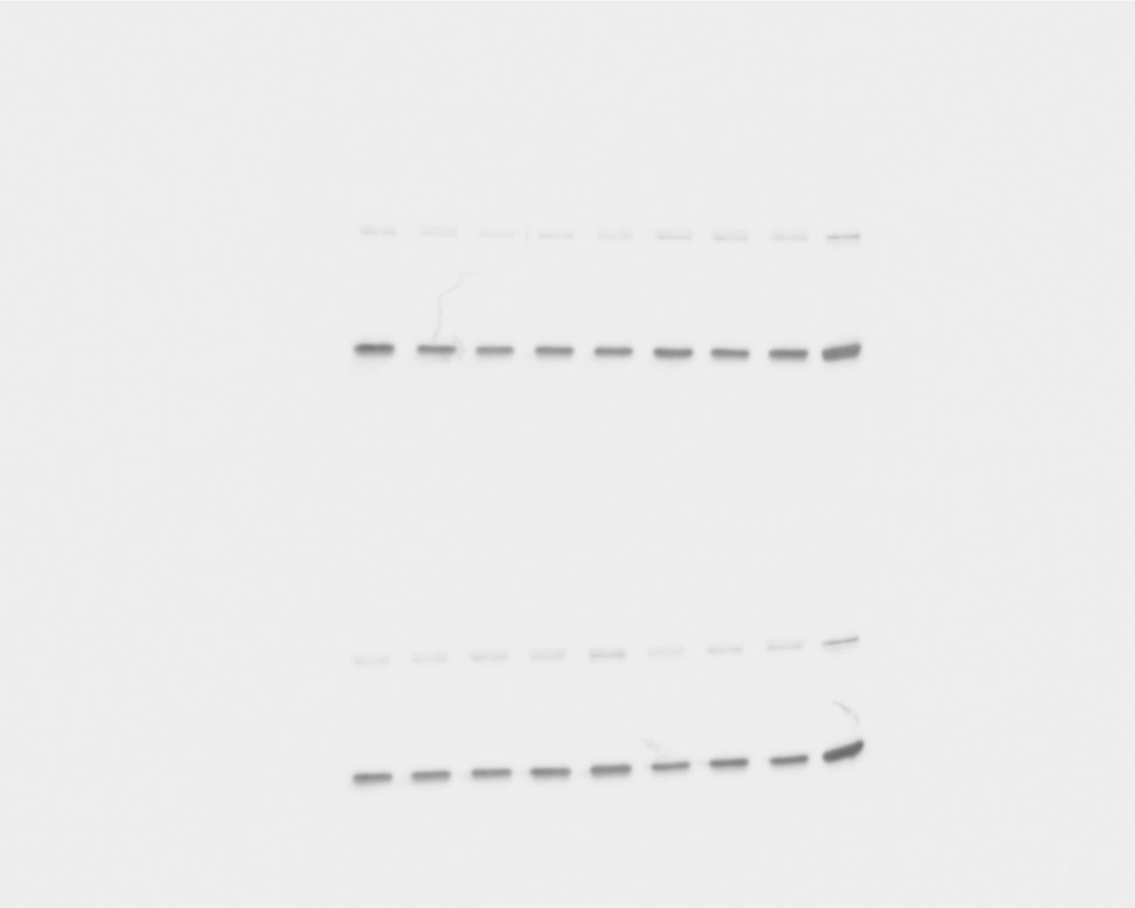

Exposition time = 11 seconds

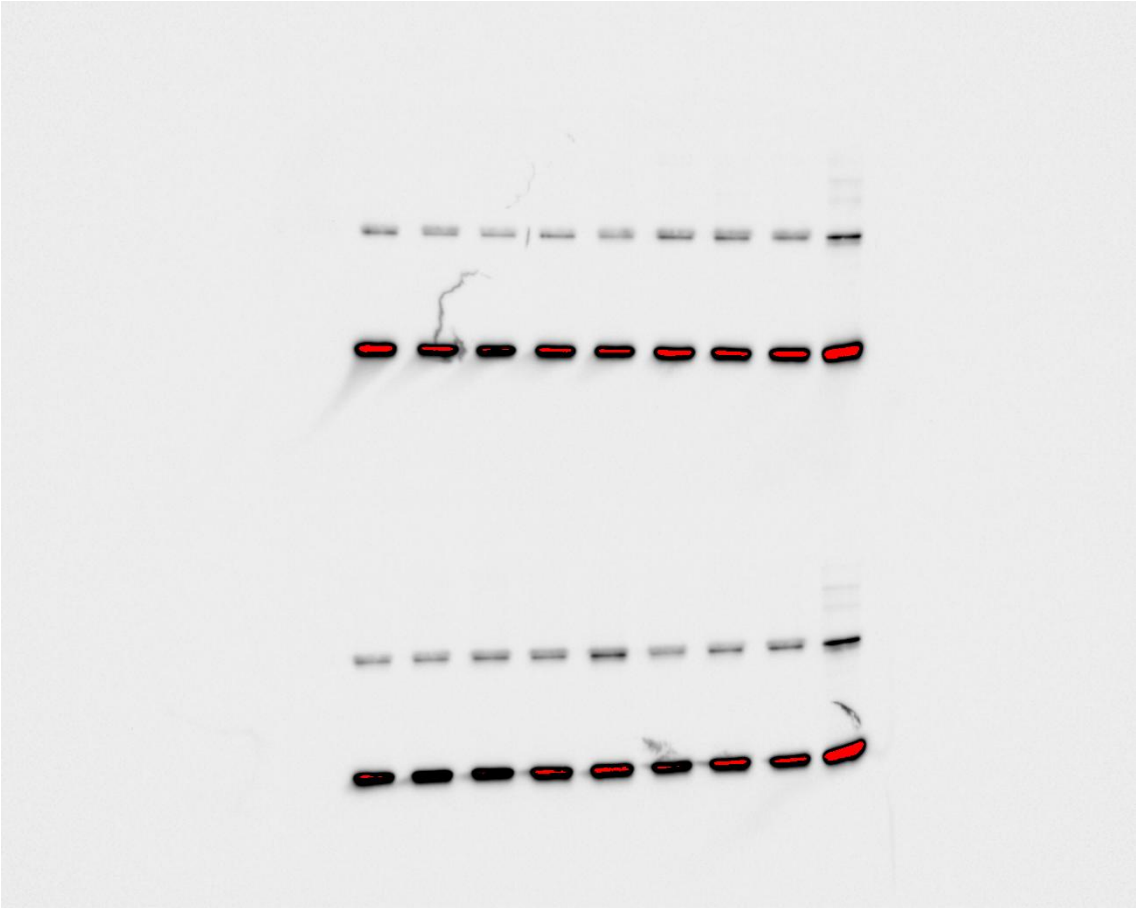

Blot 5 and 6

Blot 5	MW	1 CSF NC M	2 CSF NC M	3 CSF NC M	4 CSF NC M	5 CSF Dem.-AD M	6 CSF Dem.-AD M	7 CSF Dem.-AD M	8 CSF Dem.-AD M	recTTR 100 ng
Blot 6	MW	1 CSF MCI-AD M	2 CSF MCI-AD M	3 CSF MCI-AD M	4 CSF MCI-AD M	5 CSF MCI-AD M	6 CSF Dem.-AD M	7 CSF Dem.-AD M	8 CSF Dem.-AD M	recTTR 100 ng

Exposition time = 1 second

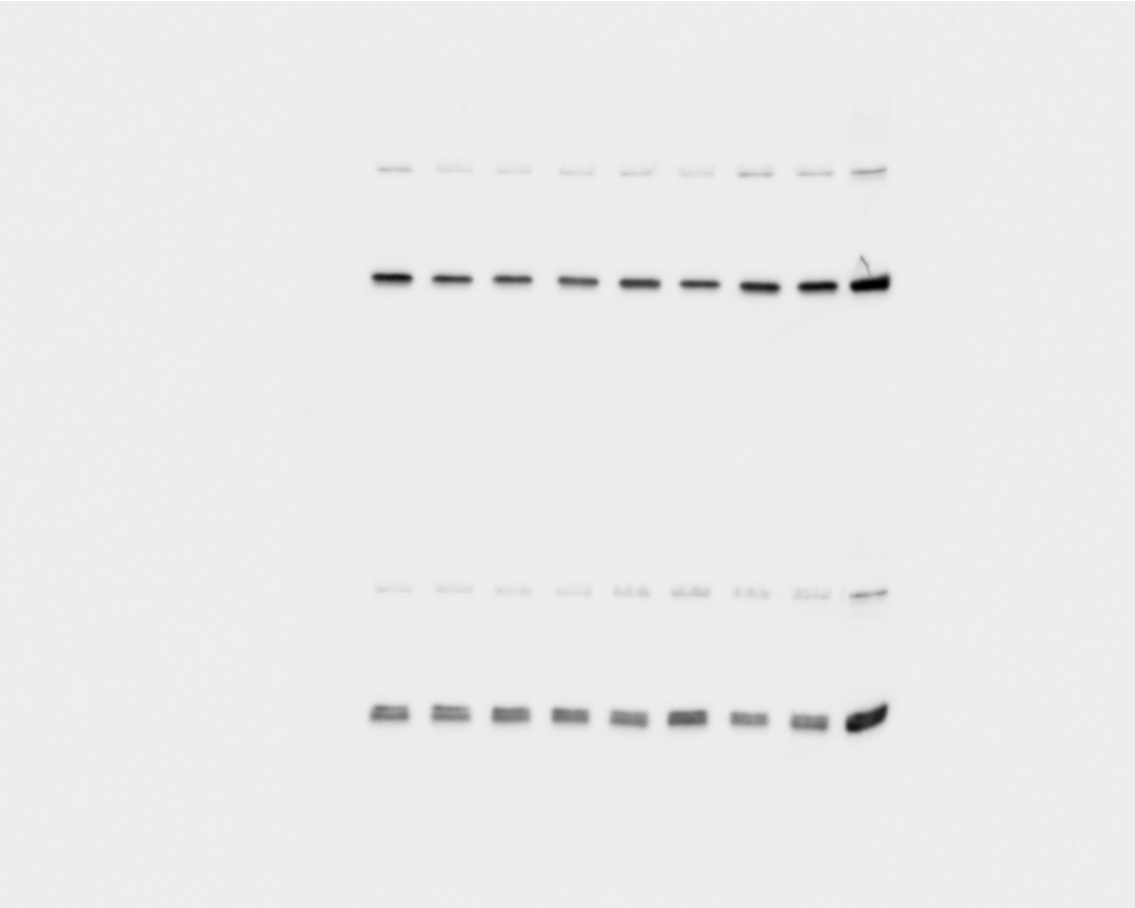

Exposition time = 11 seconds

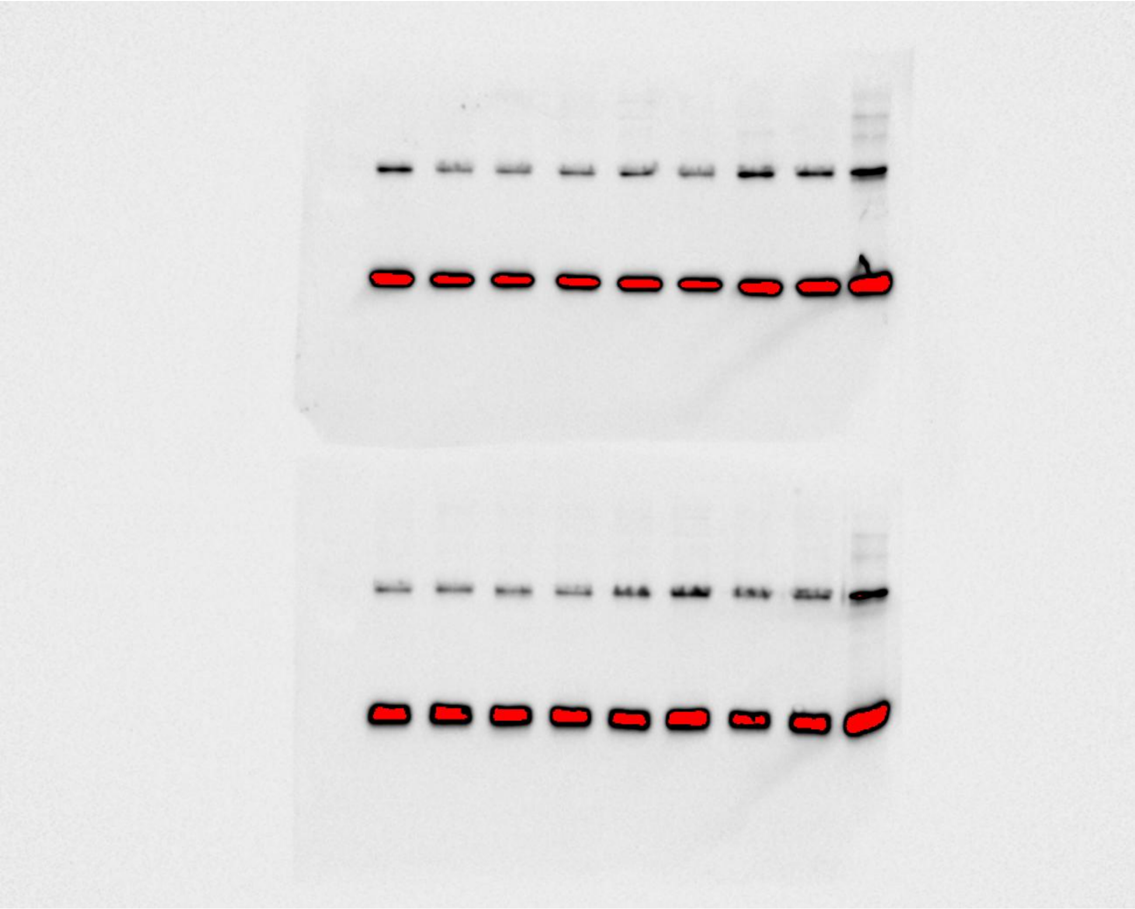

Blot 7 and 8

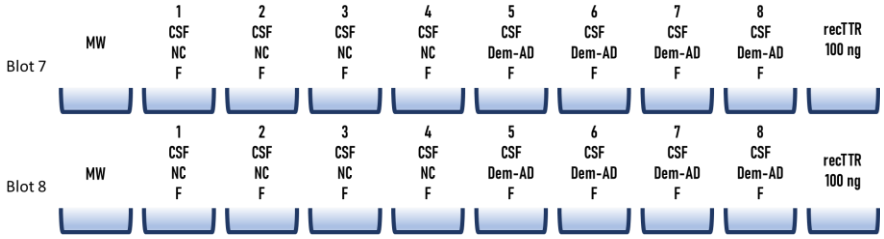

Exposition time = 1 second

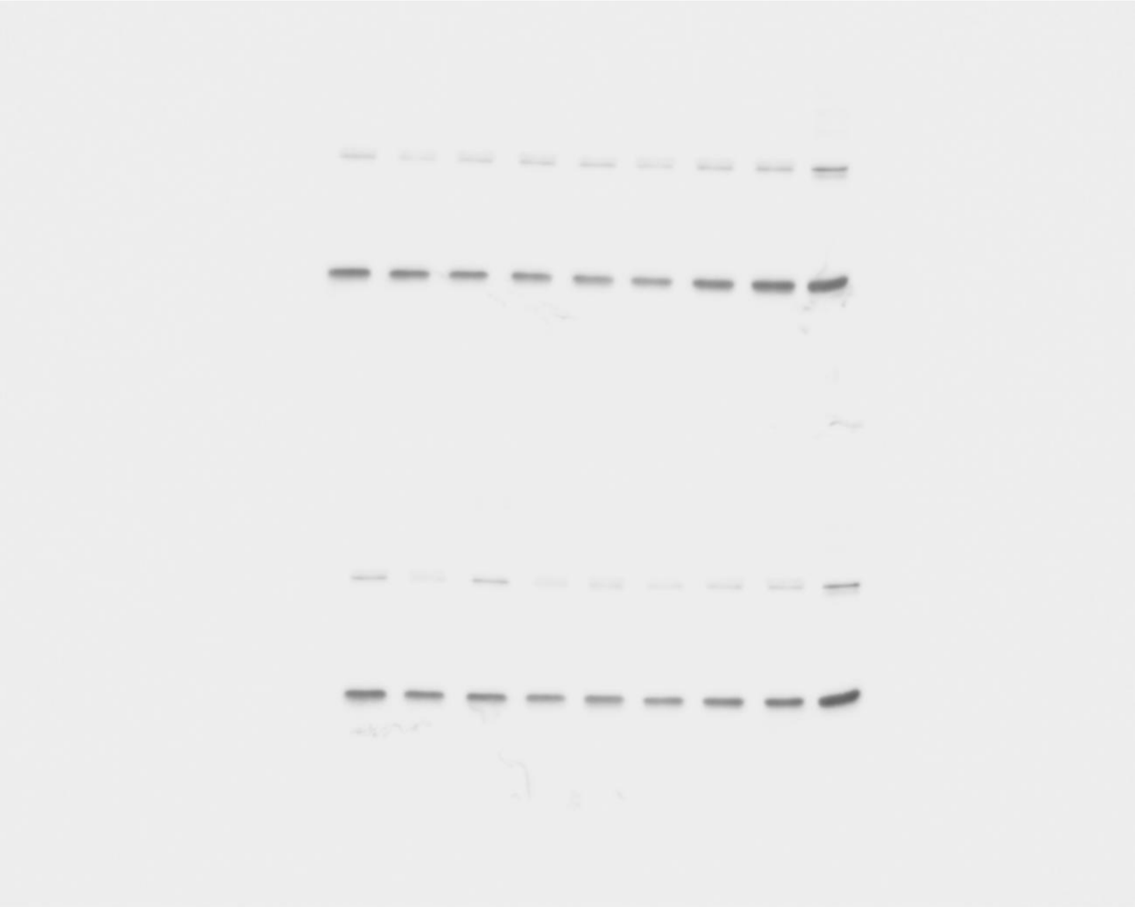

Exposition time = 11 seconds

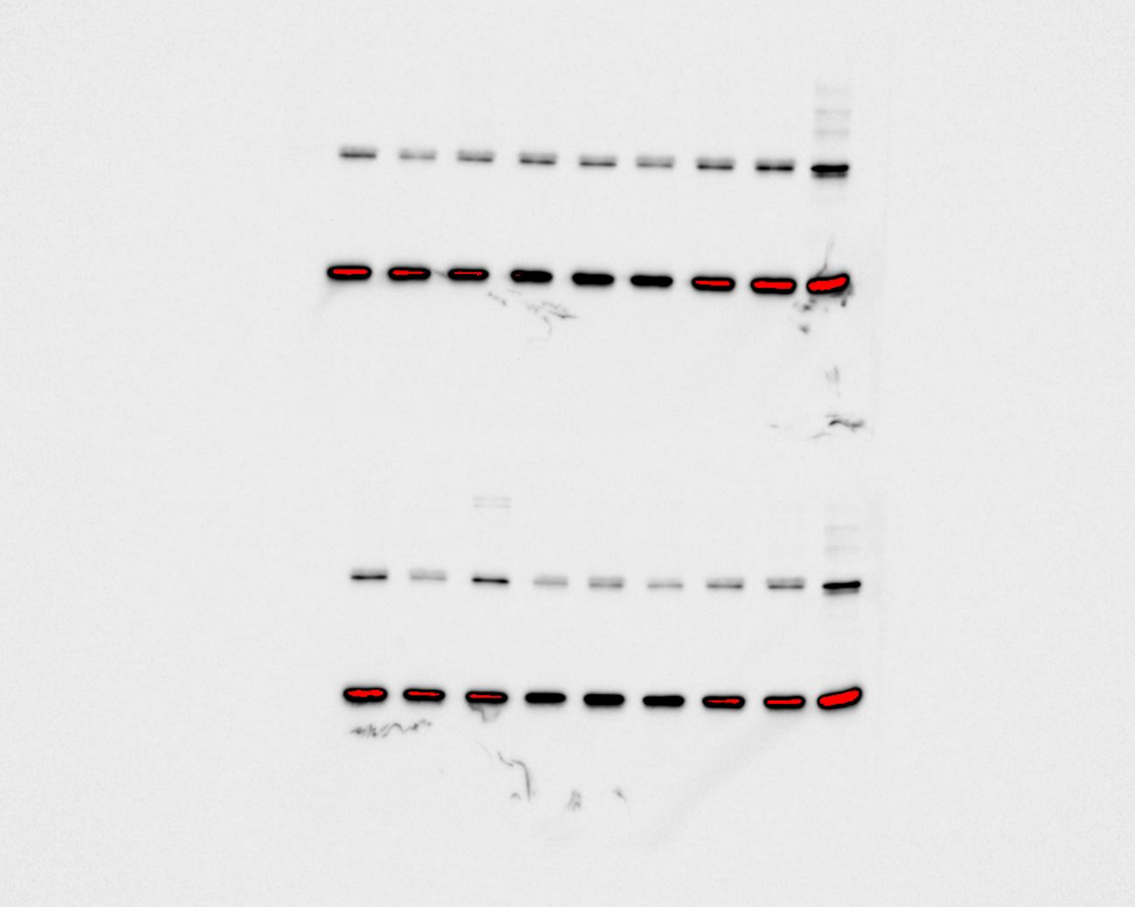

Blot 9 and 10

	MW	1 CSF MCI-AD F	2 CSF MCI-AD F	3 CSF MCI-AD F	4 CSF MCI-AD F	5 CSF Dem-AD F	6 CSF Dem-AD F	7 CSF Dem-AD F	8 CSF Dem-AD F	recTTR 100 ng
Blot 9										

	MW	1 CSF NC F	2 CSF NC F	3 CSF MCI-AD F	4 CSF MCI-AD F	5 CSF MCI-AD F	6 CSF MCI-AD F	7 CSF MCI-AD F	8 CSF MCI-AD F	recTTR 100 ng
Blot 10										

Exposition time = 1 second

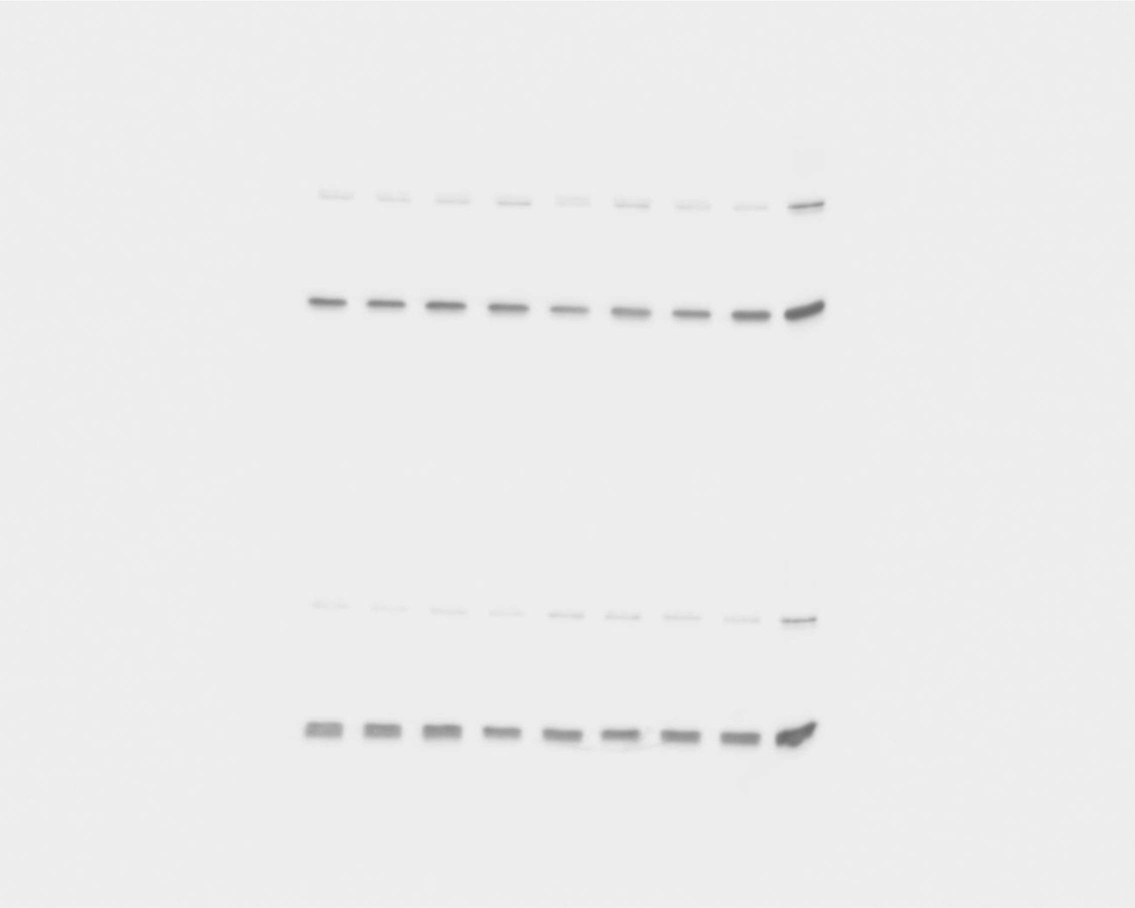

Exposition time = 11 seconds

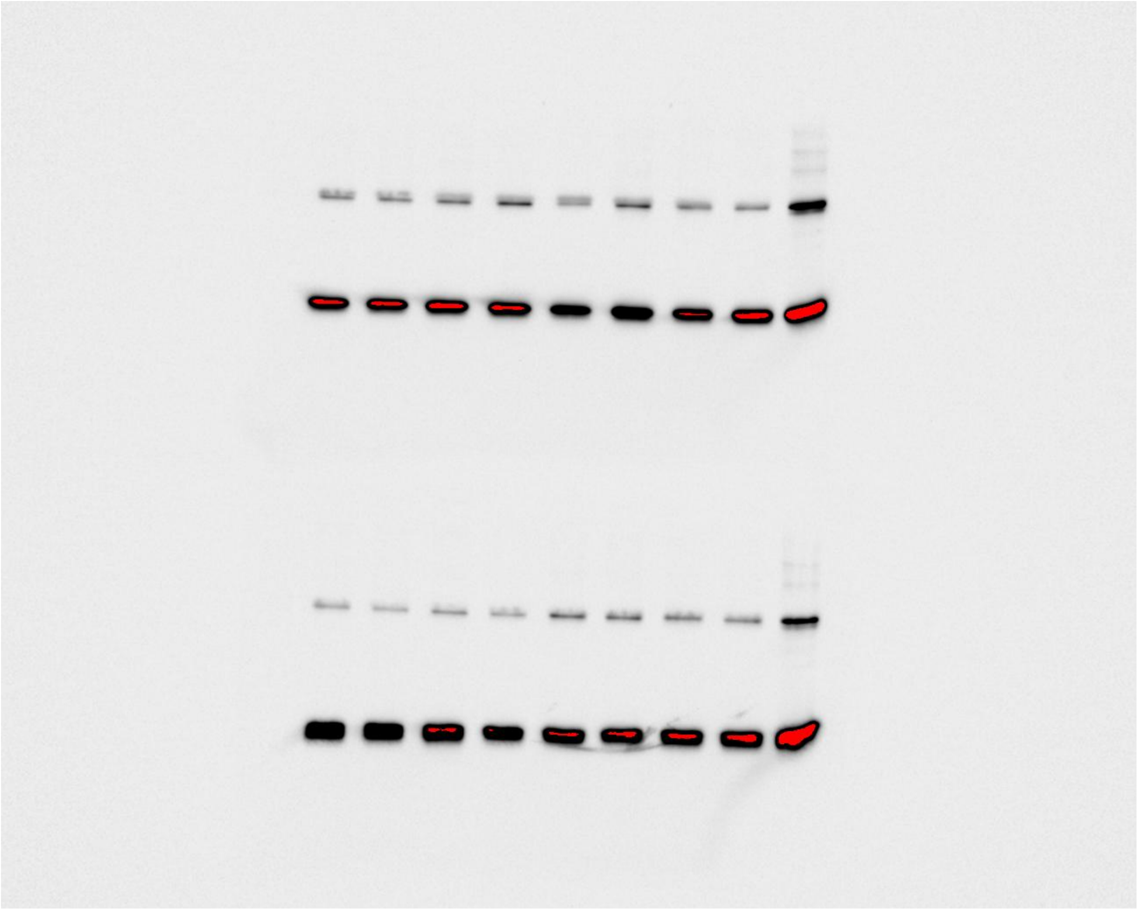

Blot 11 and 12

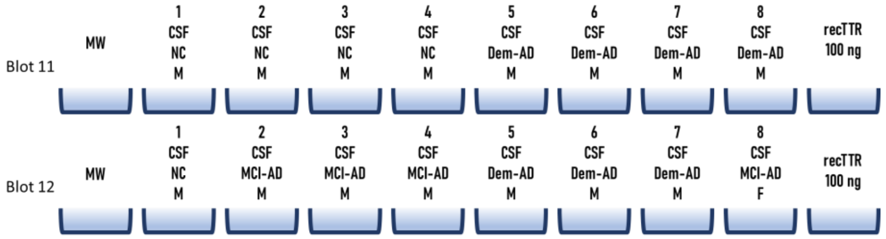

Exposition time = 1 second

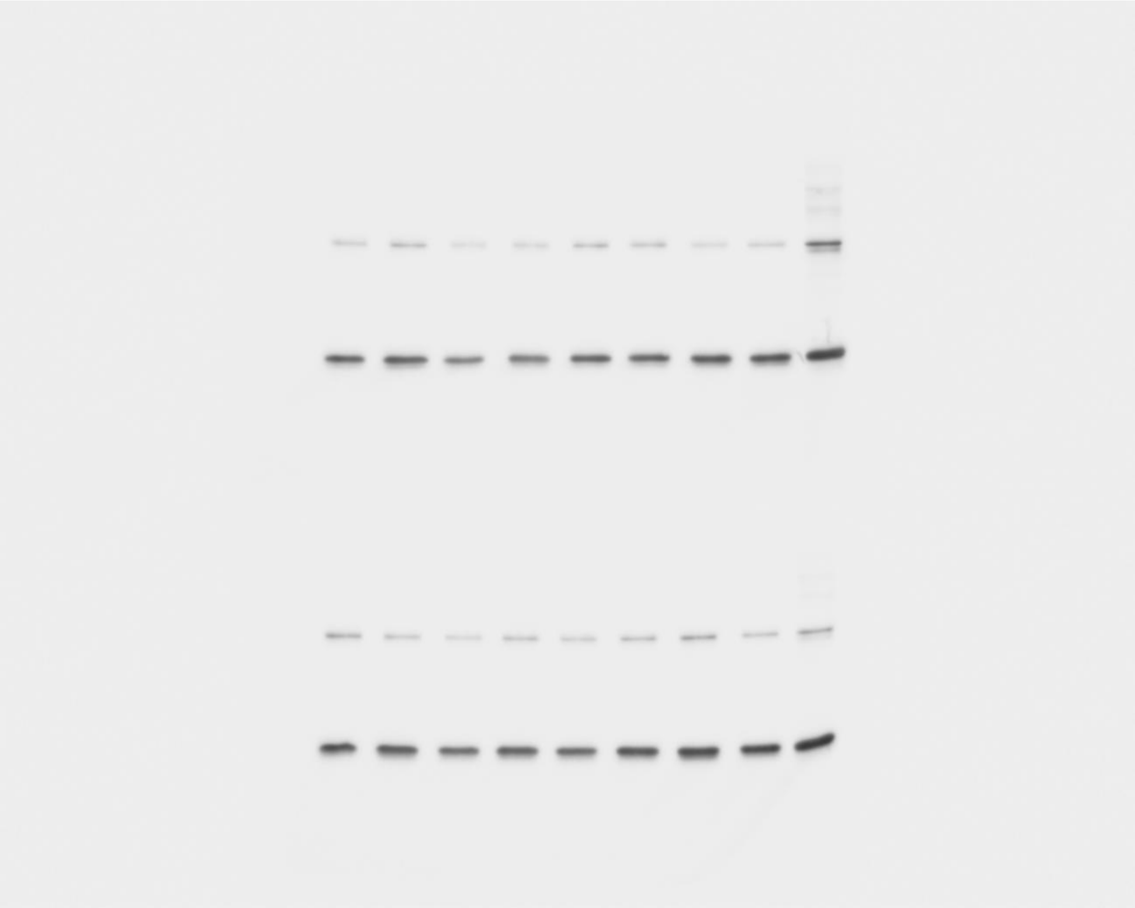

Exposition time = 11 seconds

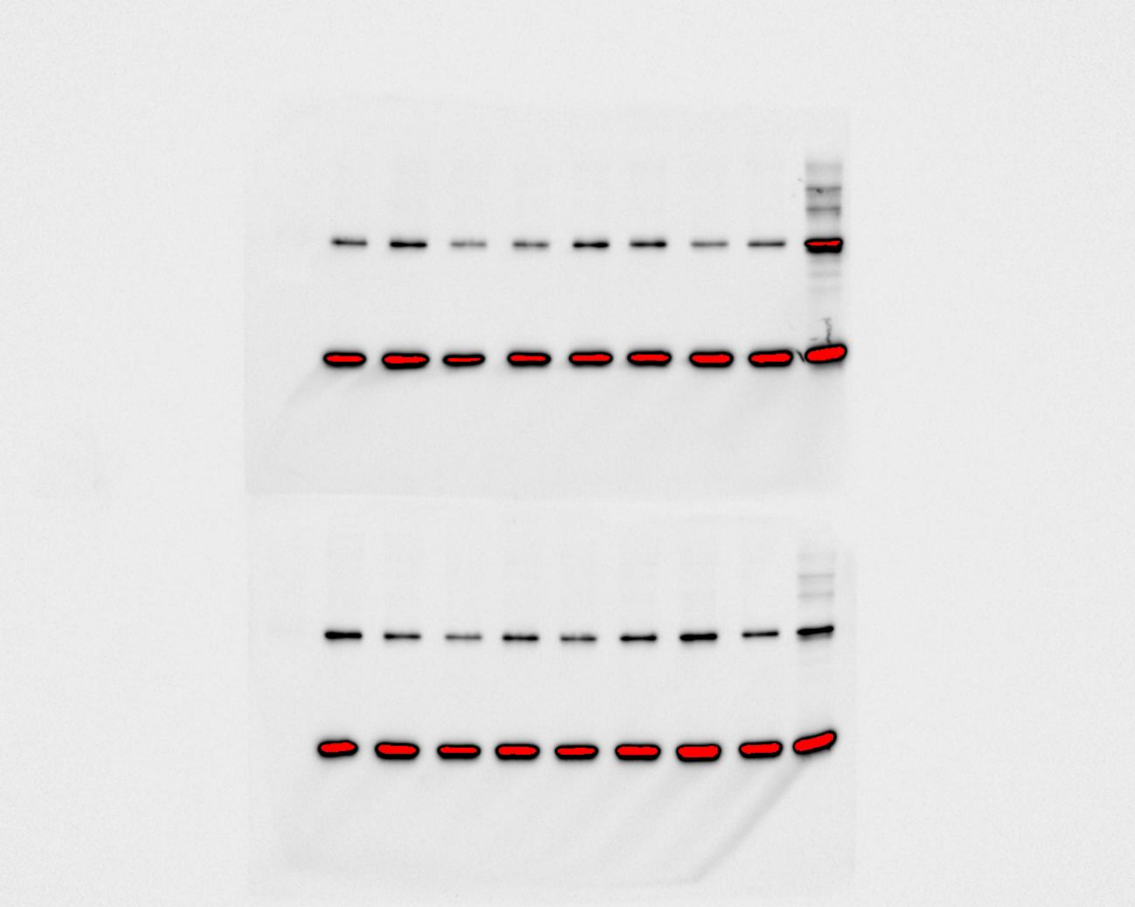

2.2. Evaluation of CSF instability – 2nd set of blots (12 blots)

Blot 1 and 2

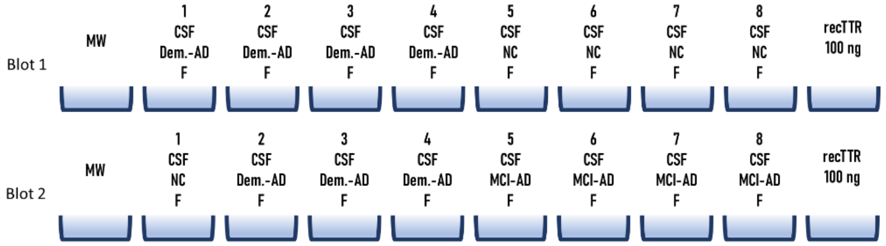

Exposition time = 1 second

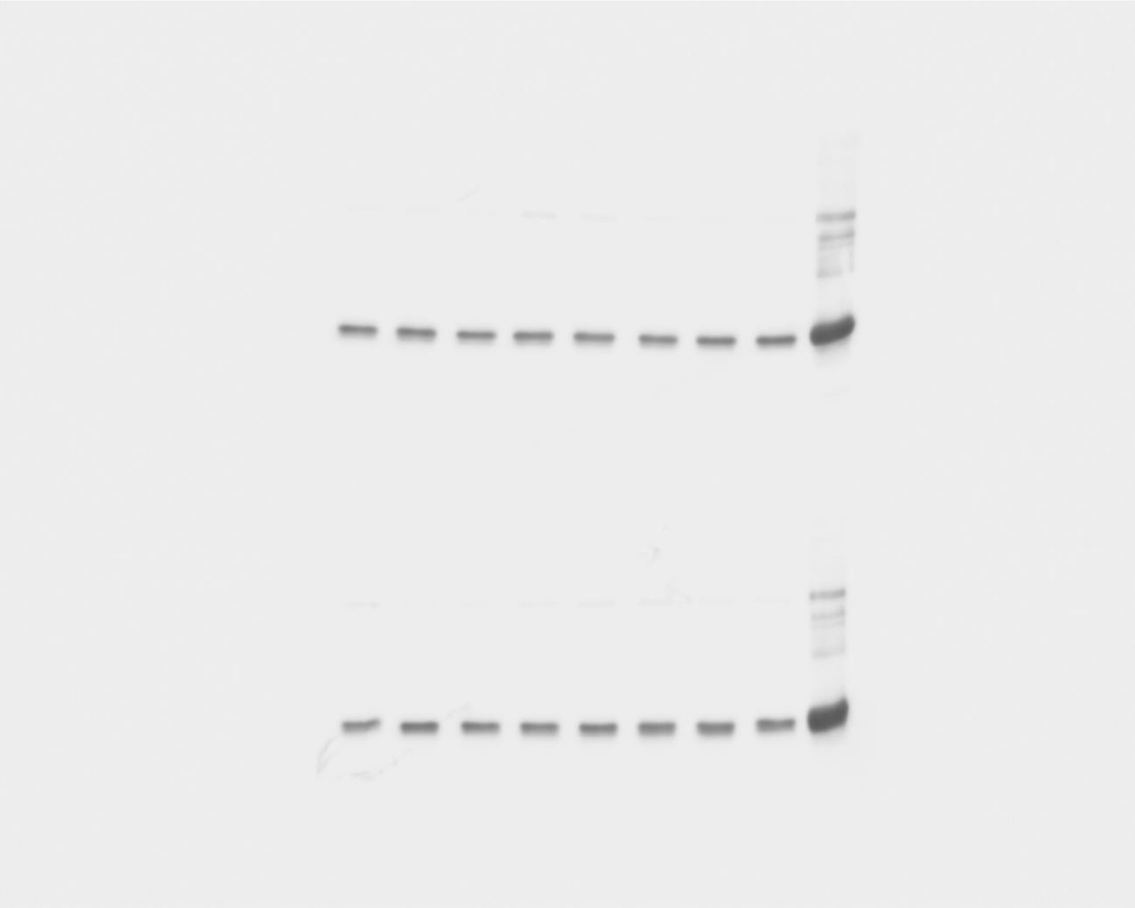

Exposition time = 11 seconds

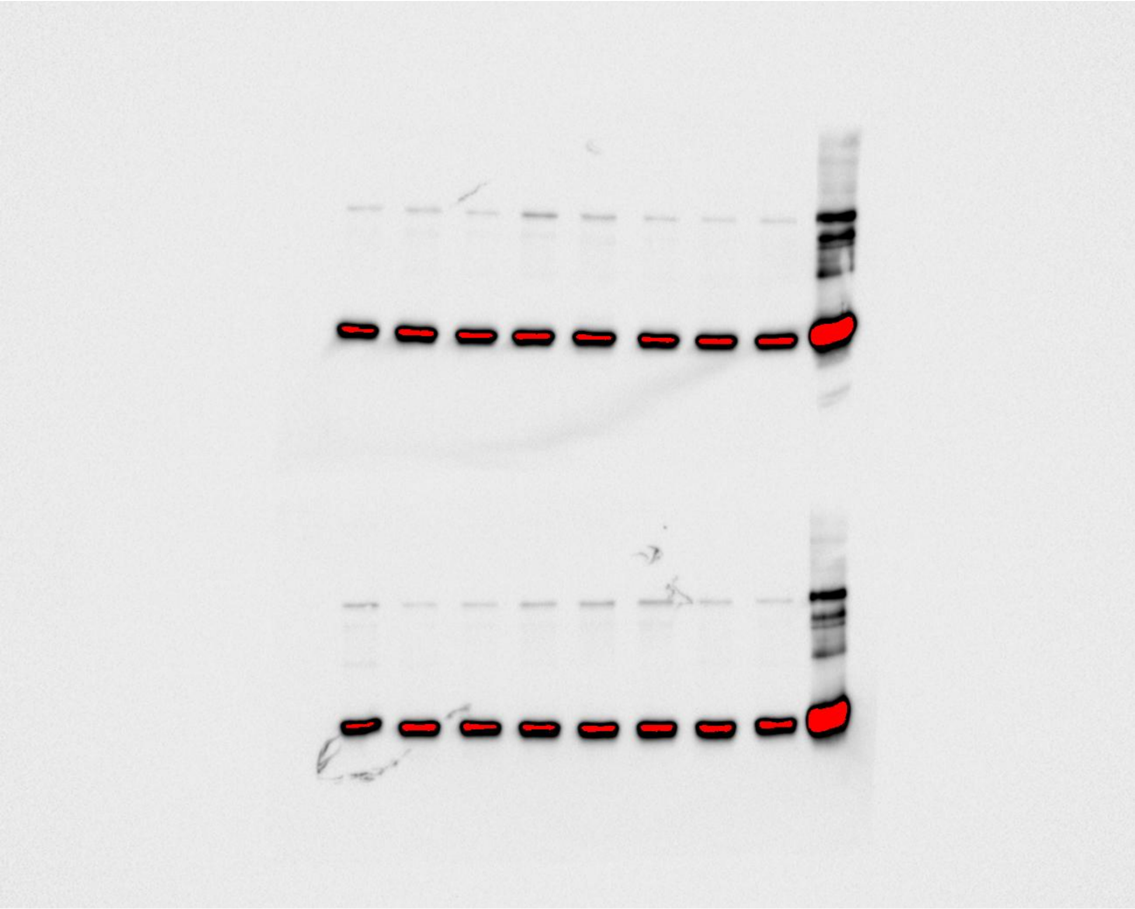

Blot 3 and 4

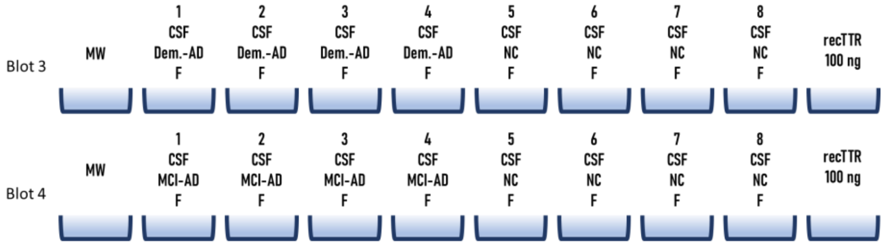

Exposition time = 1 second

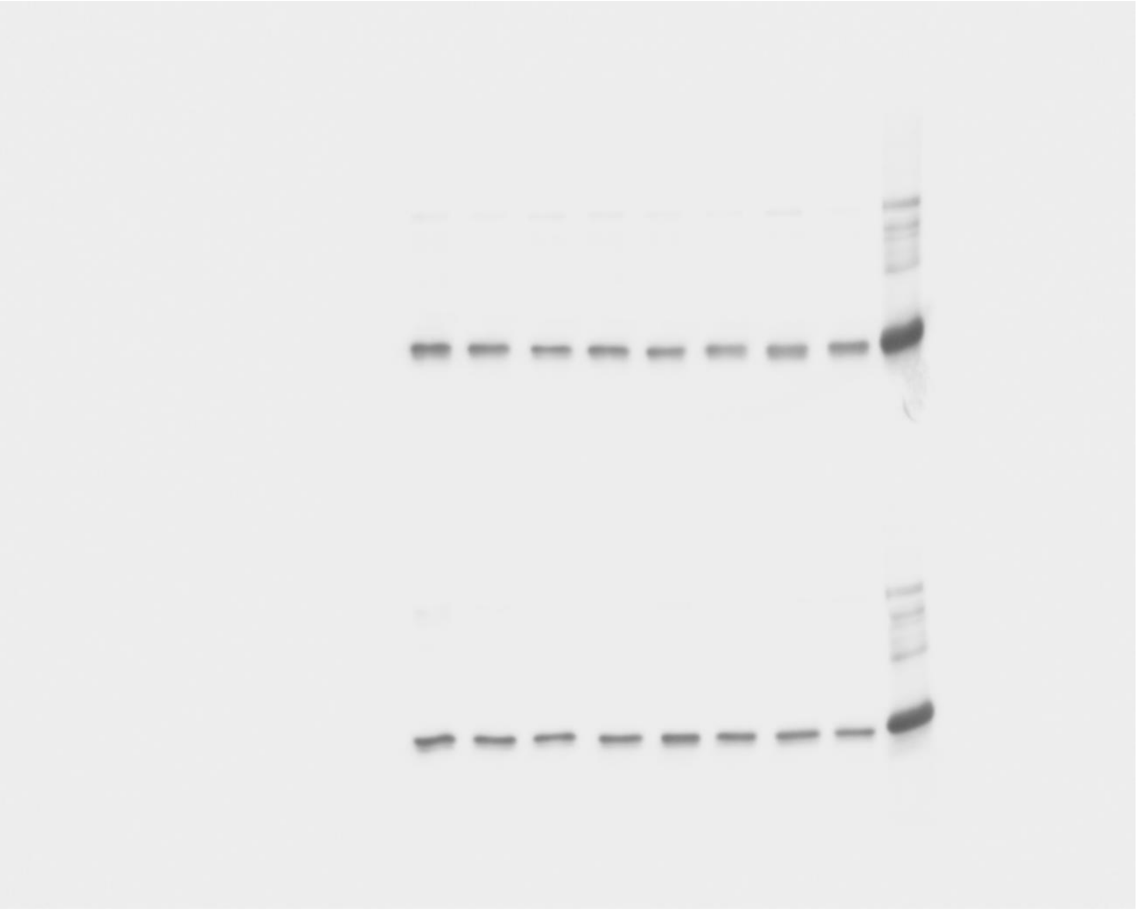

Exposition time = 11 seconds

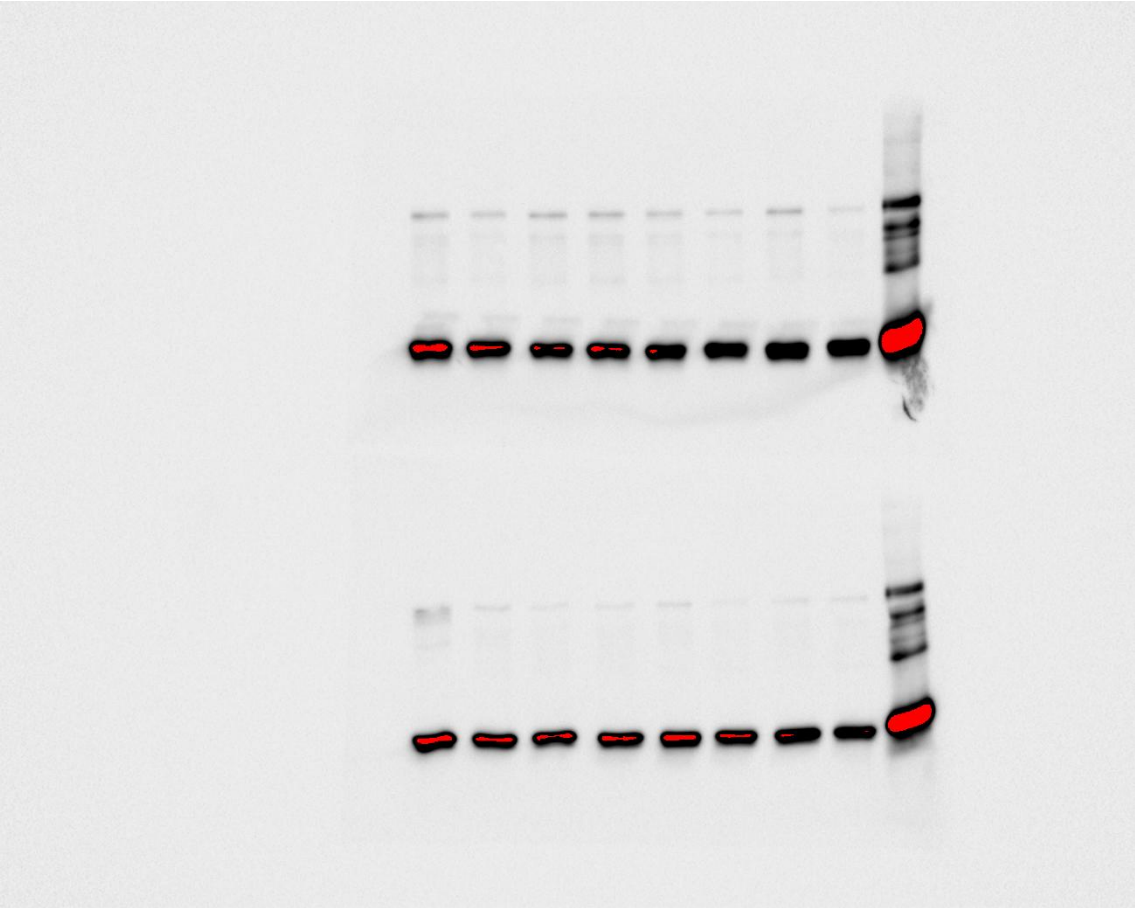

Blot 5 and 6

Blot 5	MW	1 CSF MCI-AD F	2 CSF MCI-AD F	3 CSF MCI-AD F	4 CSF MCI-AD F	5 CSF NC F	6 CSF NC F	7 CSF NC F	8 CSF NC F	recTTR 100 ng
Blot 6	MW	1 CSF Dem.-AD F	2 CSF Dem.-AD F	3 CSF Dem.-AD F	4 CSF Dem.-AD F	5 CSF MCI-AD F	6 CSF MCI-AD F	7 CSF MCI-AD F	8 CSF MCI-AD F	recTTR 100 ng

Exposition time = 1 second

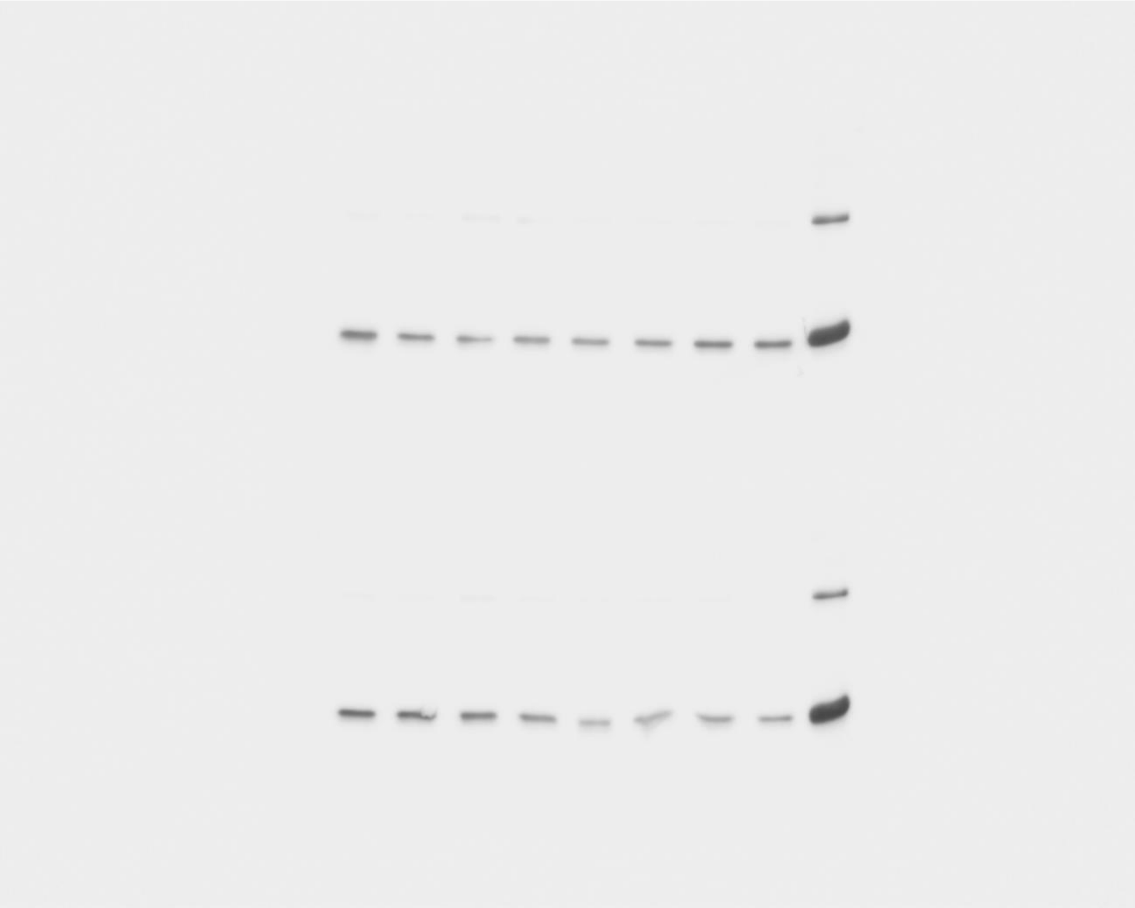

Exposition time = 11 seconds

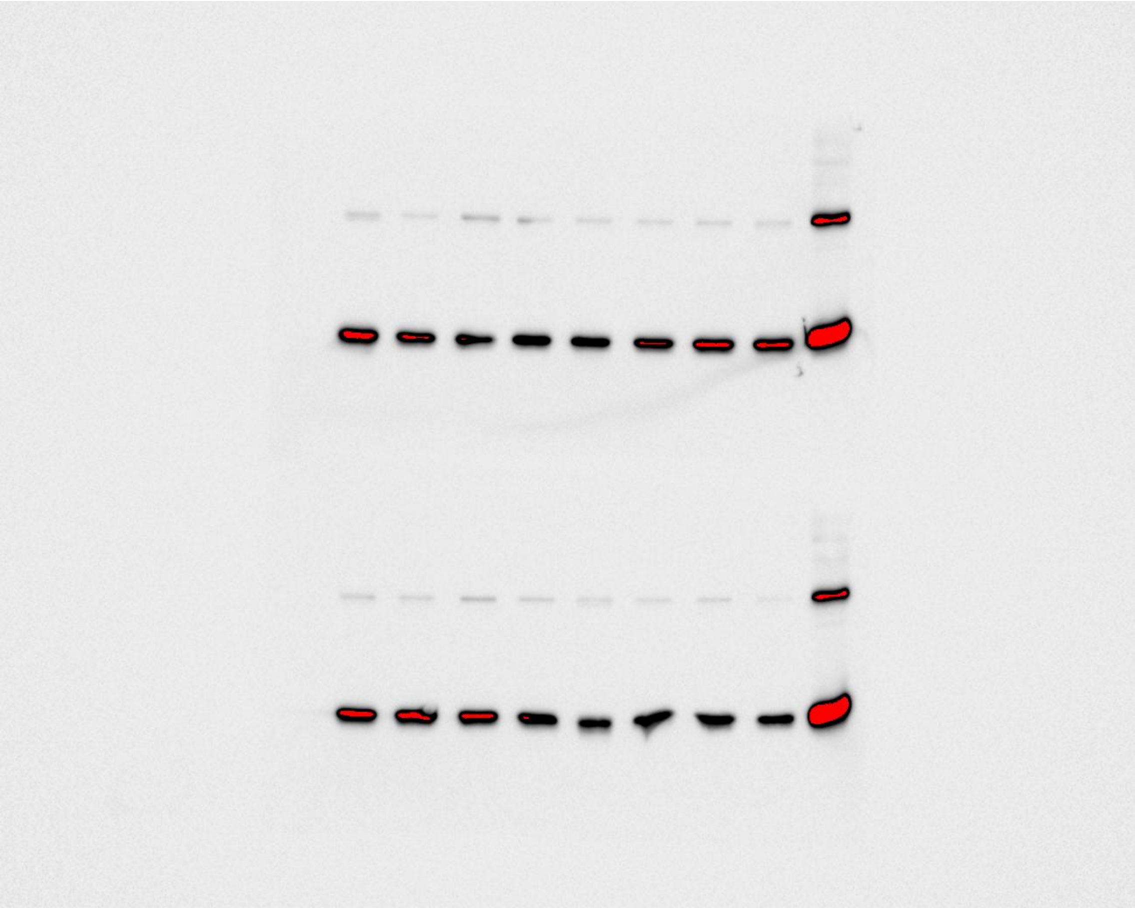

Blot 7 and 8

Blot 7	MW	1 CSF Dem.-AD F	2 CSF Dem.-AD F	3 CSF Dem.-AD F	4 CSF Dem.-AD F	5 CSF MCI-AD F	6 CSF MCI-AD F	7 CSF MCI-AD F	8 CSF MCI-AD F	recTTR 100 ng
Blot 8	MW	1 CSF Dem.-AD F	2 CSF Dem.-AD F	3 CSF Dem.-AD F	4 CSF Dem.-AD F	5 CSF NC F	6 CSF Dem.-AD M	7 CSF Dem.-AD M	8 CSF MCI-AD M	recTTR 100 ng

Exposition time = 1 second

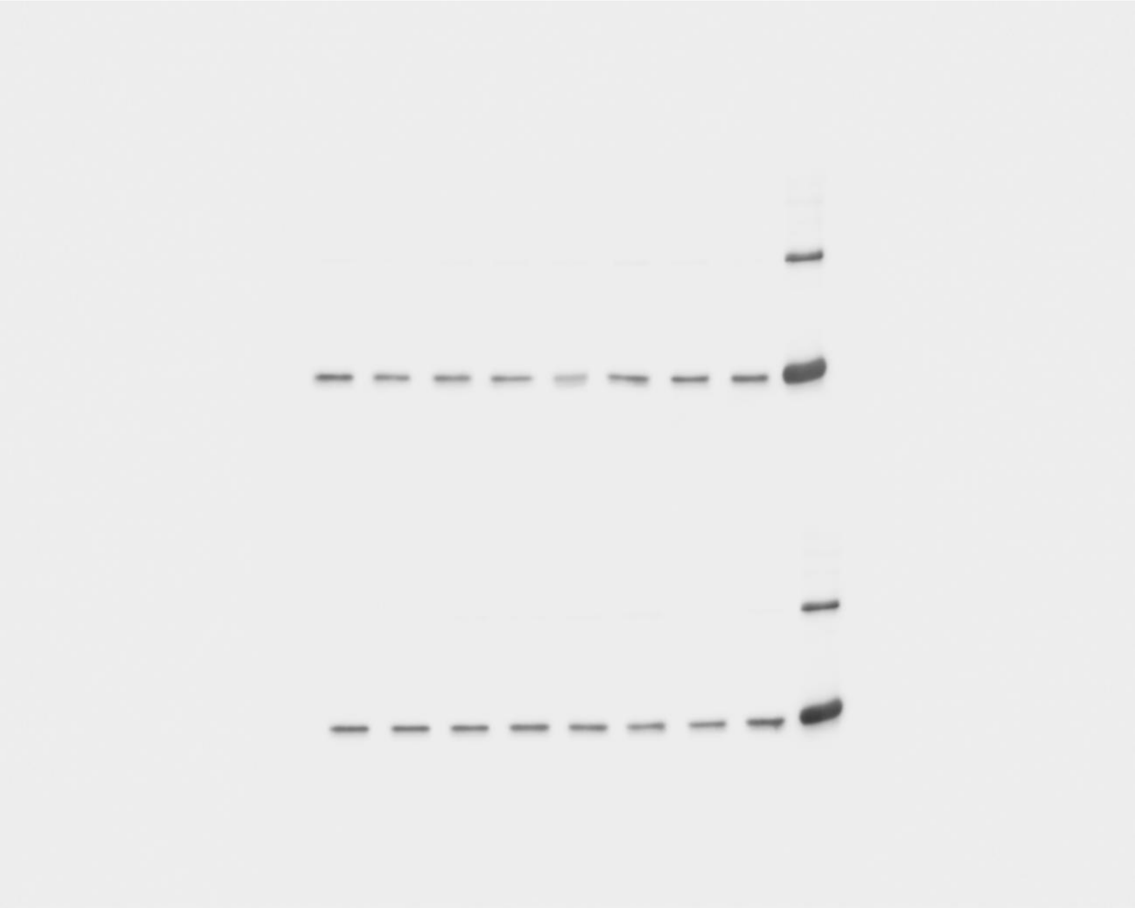

Exposition time = 11 seconds

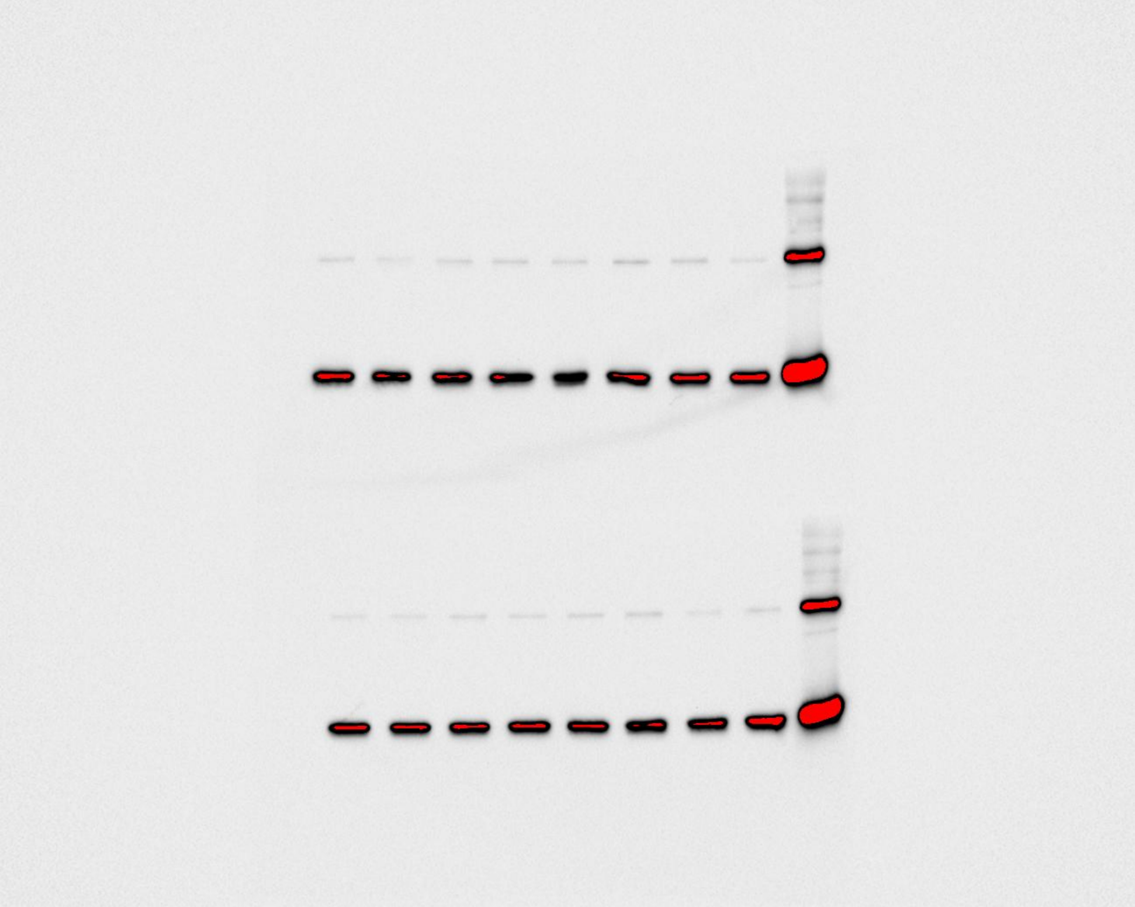

Blot 9 and 10

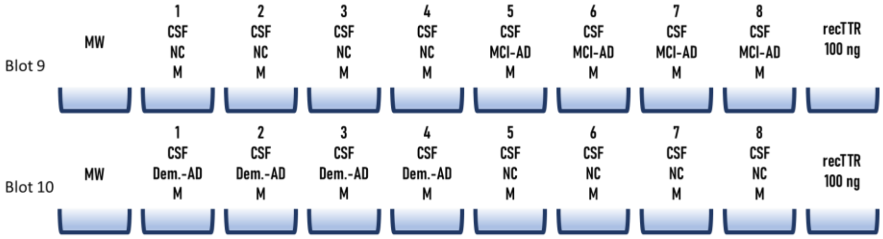

Exposition time = 1 second

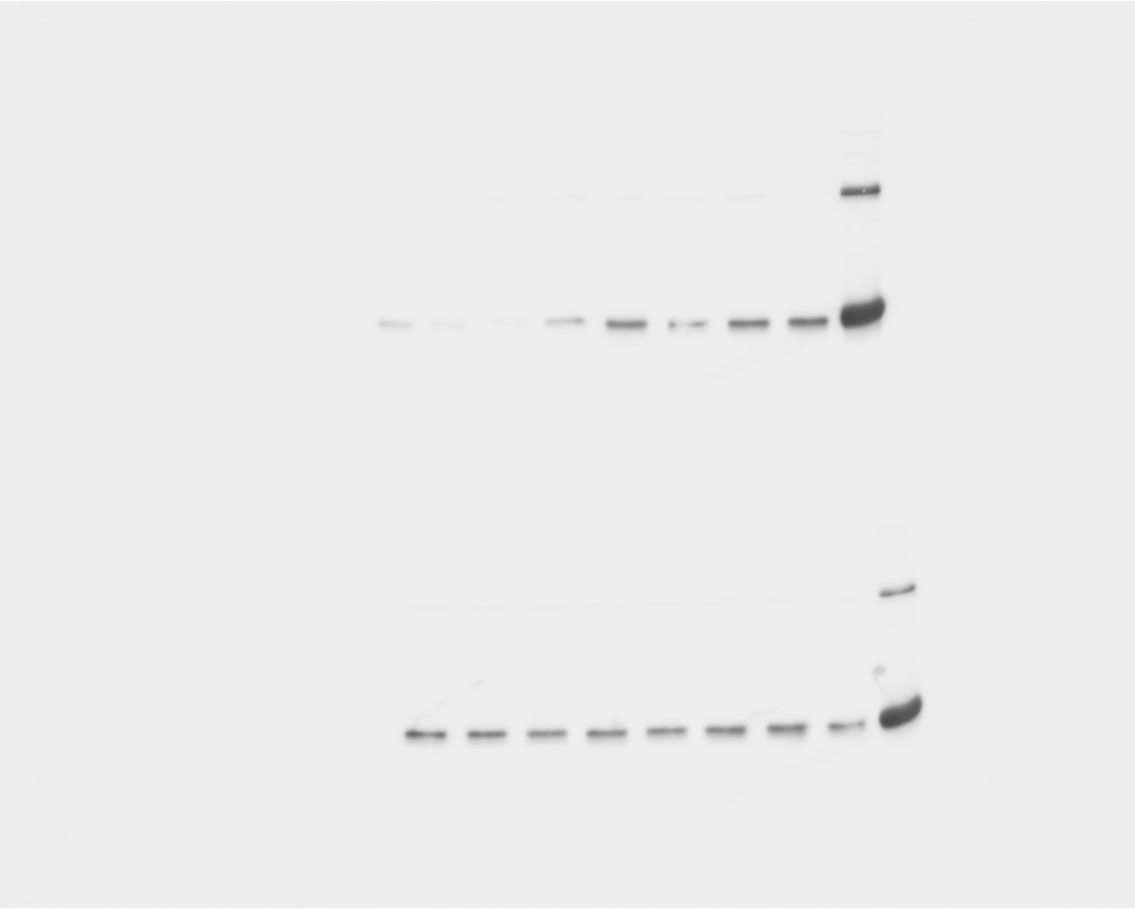

Exposition time = 11 seconds

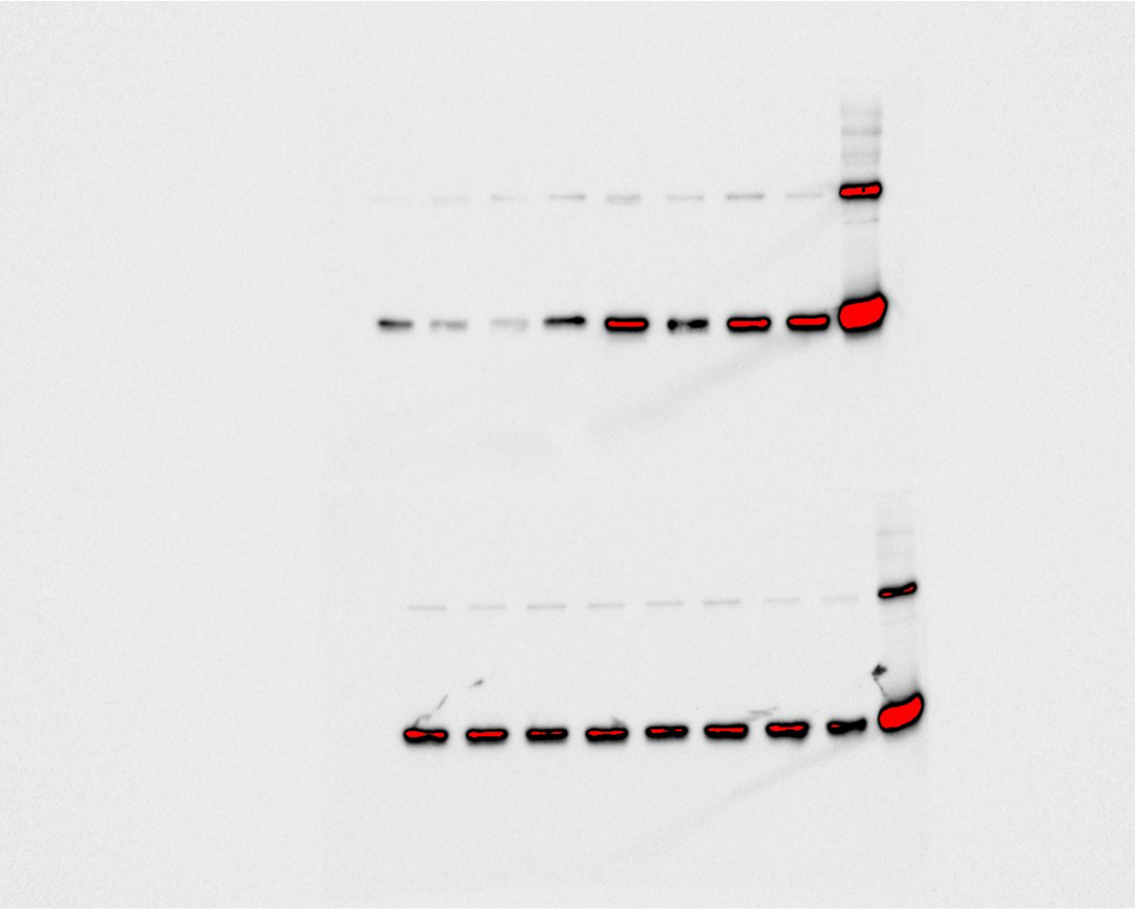

Blot 11 and 12

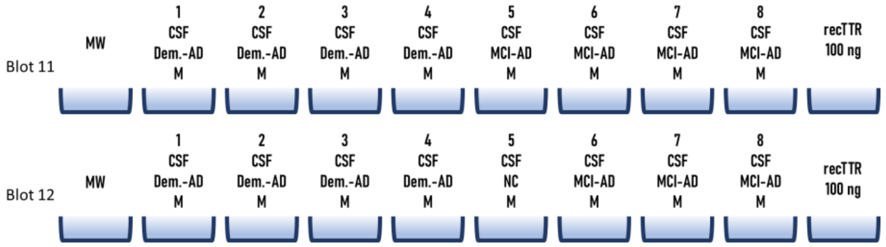

Exposition time = 1 second

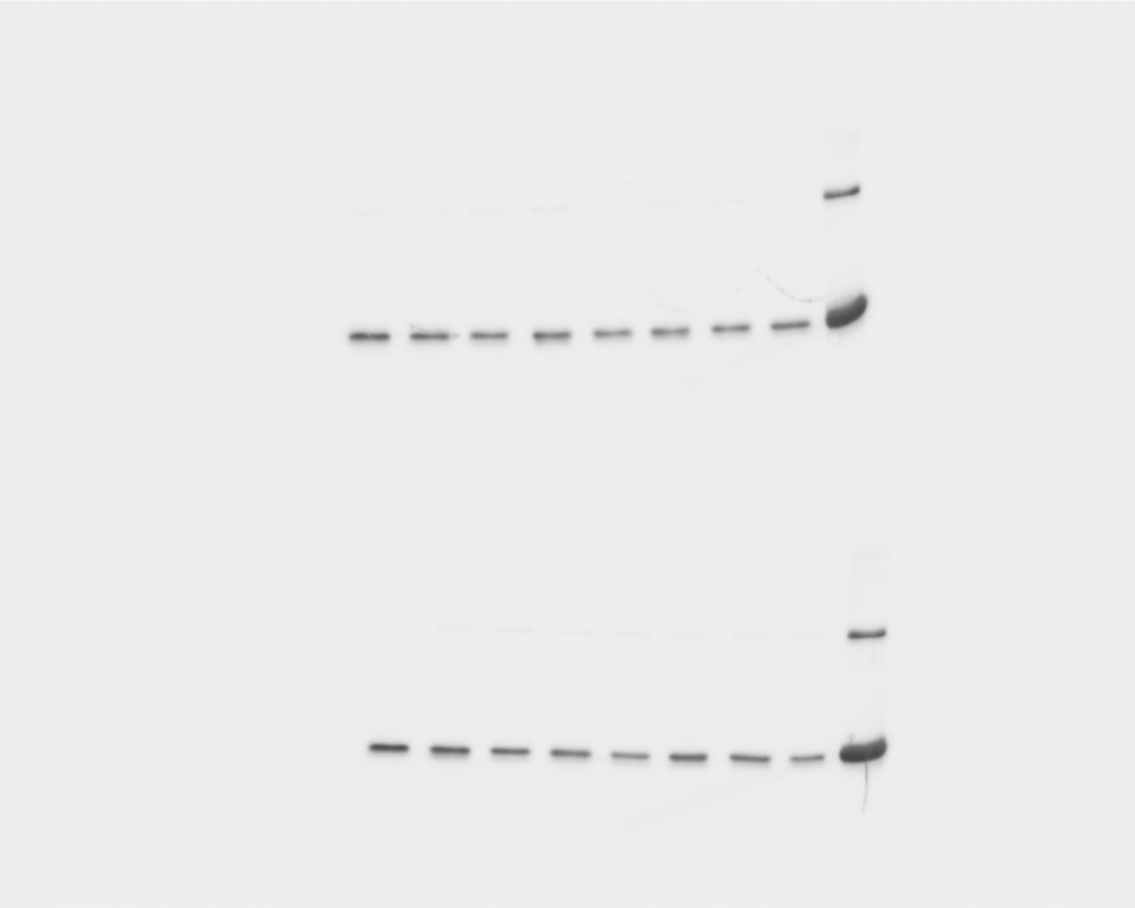

Exposition time = 11 seconds

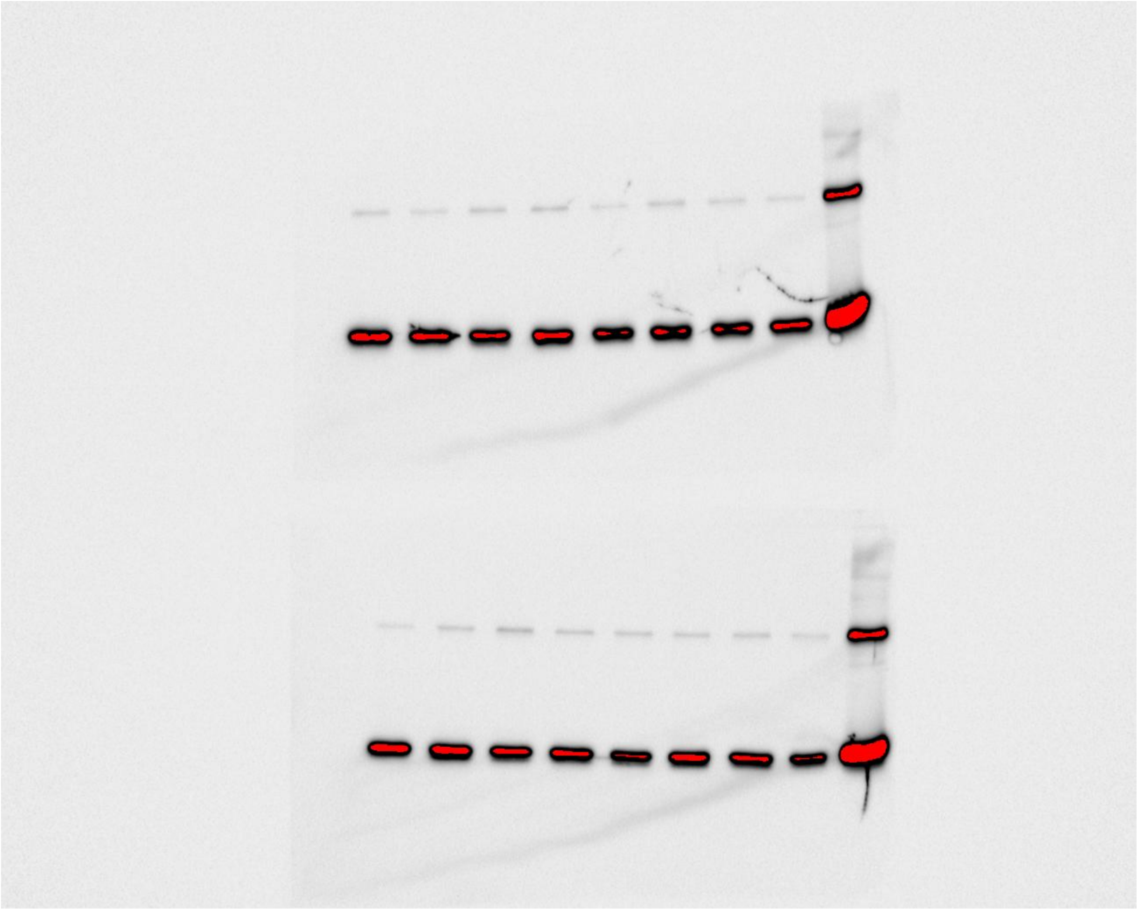

3. Evaluation of the effect of TTR binding to Aβ42 peptide

The image shows the uncropped blot presented in Figure 4.

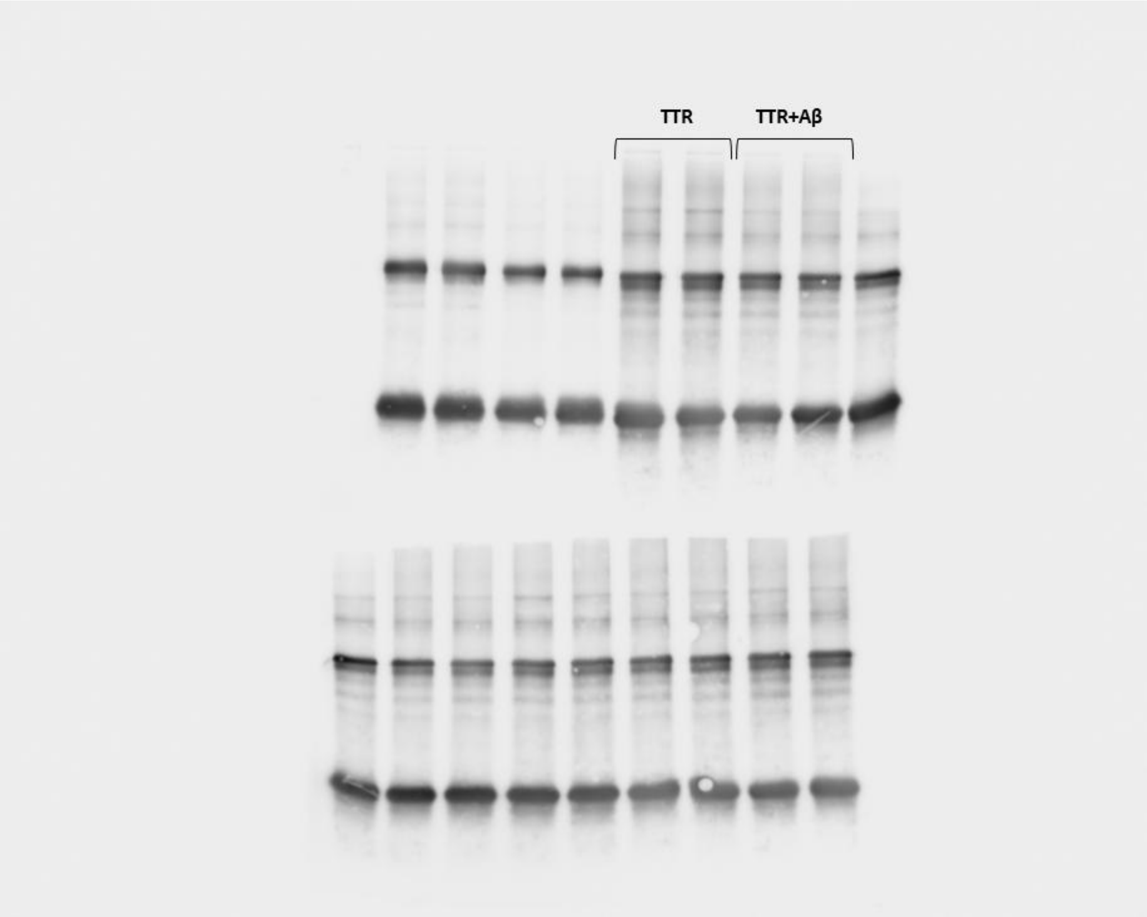

Supplement: Supplementary file 1 — Supplementary Material 1 [file 41598_2026_41717_MOESM1_ESM.pdf]
